# Cross-cultural comparison of nudging effects for environmental protection: A case-study of risk-averse attitudes toward disposable plastics

**DOI:** 10.1371/journal.pone.0277183

**Published:** 2022-11-03

**Authors:** Hidenori Komatsu, Hiromi Kubota, Nobuyuki Tanaka, Mariah Griffin, Jennifer Link, Glenn Geher, Maryanne L. Fisher

**Affiliations:** 1 Grid Innovation Research Laboratory, Central Research Institute of Electric Power Industry, Yokosuka, Kanagawa, Japan; 2 Sustainable System Research Laboratory, Central Research Institute of Electric Power Industry, Abiko, Chiba, Japan; 3 Department of Psychology, State University of New York at New Paltz, New Paltz, New York, United States of America; 4 Animal Behavior Graduate Group, University of California, Davis, Davis, California, United States of America; 5 Faculty of Science, Department of Psychology, Saint Mary’s University, Halifax, Nova Scotia, Canada; Shandong University of Science and Technology, CHINA

## Abstract

Disposable plastics are drawing considerable attention as a source of environmental risk despite their benefits in daily life. Banning the use of disposable plastics could increase other types of risks, which may damage the public good in the long run. Considering the trade-off of the risks and benefits, one way to improve social welfare is to conduct proper recycling and to continue using plastics but limit them to essential use, avoiding an unnecessary ban. A potential barrier to such a policy might be risk-averse attitudes toward actions that are perceived to threaten future generations, which is a well-known phenomenon. We previously designed a framework for information provision using messages that remind individuals about familial support, which had significant effects in multiple countries on increasing positive attitudes toward air pollution caused by industrialization. We hypothesized that this information provision could also be effective for disposable plastic use. Thus, we conducted a randomized controlled trial via online surveys in Japan, Canada, and the US to identify the effects of our designed messages about recycling on increasing positive attitudes toward disposable plastics. The intervention effects were measured by the difference-in-difference method and panel analysis based on linear regression models using the respondents’ attributes and personality traits. The effects were consistently correlated with a sense of familial support, with the effect sizes varying according to country (US > Japan > Canada). Attributes that positively contributed to the message being more effective were higher agreeableness, lower Machiavellianism, lower psychopathy, and being a woman. Although personal fear about COVID-19 moderated the message effects, concern about the threats to relatives and family boosted the effects. Although the effect sizes were influenced by external factors, the results suggested that our proposed framework for information provision has the potential to be applied to a wider variety of risk-related topics.

## Introduction

Disposable plastic waste is drawing considerable attention from researchers and the media, mainly in the context of how it leads to environmental problems [1, 2]. Correspondingly, many countries have created policies to reduce plastic garbage. In 2020, Japan started an economic intervention to begin charging for plastic bags in supermarkets or convenience stores [3] and enacted a law to promote reducing and recycling plastics in 2022 [4]. Canada officially declared a nation-wide policy to ban disposable plastic use in 2019 [5]. In the US, three states have banned plastic use already and 10 states have legislation to ban the use of plastic bags [6], but the country remains the top generator of disposable plastics [7]. The European Union enacted a regulation to ban 10 types of single-use plastic products in 2019 [8]. Although the current levels of legislation are different across countries or local governments, the global trend is that the regulations are shifting toward banning the use of disposable plastics [9].

The use of plastics has benefited our daily life by improving consumer health through packaging for food, medical supplies, and other personal goods to reduce contamination [10]. Another benefit is the energy savings in transportation compared with packaging that relies on heavier materials [11]. Nonetheless, plastic use is currently subject to severe criticism, especially in the context of Sustainable Development Goals (SDGs) [12, 13]. Risk-averse attitudes toward disposable plastics are observed, which could be a driving force for promoting unrealistic or needless public policy. For example, although Japan created a policy to charge for plastic bags, these bags are only 2% of all the plastic garbage that has drifted ashore in Japan [14], suggesting that the policy intervention effects are almost negligible even if all plastic bag use were ended. There are unintended consequences of banning plastic bag use, such as increases in paper bag consumption [15], increased purchase transaction duration [16], or a shift toward the purchase of larger plastic bags [17]. Therefore, the potential benefits of banning disposable plastics might be less than expected and could even cause other unexpected problems. Consequently, one possibility for better social welfare could be that we continue using plastics as long as plastic recycling is properly performed, rather than simply banning all use of plastic.

Risks perceived to threaten future generations are judged as more dangerous than other types of risks [18]. Such risk-averse attitudes could influence public policy and may damage the public good, as seen in the example of dioxin regulations in Japan [19]. Furthermore, popular opinions rather than careful analysis of benefits and costs can result in policy change. The regulation for prohibiting plastic use in California was determined by popular vote [20], which suggests risk-averse attitudes can drive political decisions on the use of plastics. If a policy intervention were aimed at promoting recycling, popular risk-averse attitudes could prevent the intervention effects if the message were combined with the continuation of plastic use. Quantitative analyses for disposable plastic use have indicated that a blanket ban is less cost-effective than other alternatives [21, 22] or partly allowing the use of plastic bags [23, 24]. Similar implications have been suggested in Japan [25]. Moreover, information provision rather than regulations has been proposed as a better option considering realistic human behaviors [26]. Thus, we consider partly keeping plastic use as well as promoting information provision for environmental protection as one rational choice. In this context, designing messages to promote plastic recycling and avoid littering is a practical and important political issue.

To moderate such risk-averse attitudes, we constructed a framework for information provision. The intervention effects were identified in Japan [27] and another two countries on attitudes toward air pollution caused by industrialization (S2 Appendix in S1 File). In these previous surveys, we conducted a randomized controlled trial to investigate the effects of designed messages to remind individuals of support from older generations, compared with a basic message that described both the risks and the benefits of industrialization. The message concept was inspired by insights obtained from an evolutionary multi-agent simulation model [28], where agents join a game with a trade-off structure of risks and benefits, behave altruistically to their relatives by sharing their resources, and produce offspring over generations. The results showed that agents who were supported by relatives were more risk-prone than those who were not supported or who had many non-altruistic relatives, even if the population was risk-averse on average. This finding led to the idea that messages boosting a sense of familial support could be helpful for increasing positive attitudes in the real world. The in silico-designed intervention framework can be regarded as a nudge in the context of behavioral economics [29, 30], rooted in an evolutionary view of behavior [31, 32].

Given that a sense of familial support could increase positive attitudes toward risks that are perceived to threaten future generations, and that disposable plastics are perceived as one such risk, our proposed nudging framework could provide an effective intervention for information provision on the use of plastic. Thus, in this work, we apply a similar framework to information provision to increase positive attitudes toward disposable plastics, aimed at promoting plastic recycling. Our evolutionary simulation models suggested that receiving benefits from relatives via a risk source contributed to positive attitudes toward the risk source [28]. To convert this implication based on the simulation results into real-world interventions, the designed message needed to state that respondents had been benefiting from their relatives via disposable plastic use. Responses were obtained using an Internet questionnaire, through which a randomized controlled trial was conducted to identify the intervention effect. The surveys were conducted in Japan, Canada, and the US, similar to our previous survey on air pollution caused by industrialization (S2 Appendix in S1 File). For the cultural comparison, the intervention effects on average would not always be consistently significant and could be affected by various factors, such as social situations, even if the messages were designed based on insights extracted from biological evolution. There was wide variation in plastic use regulations across the three countries. Thus, the present study investigated social factors that could affect the size of the intervention effects as well as the effects that were rooted in altruistic evolution and common to the three countries. We achieved this by including Canada, which was an early adaptor of a nation-wide disposable plastics ban in contrast to Japan and the US, where the regulations were relatively loose. The significance of our present study is that an intervention method for promoting plastic recycling is established by customizing the message design, which showed significant effects for increasing positive attitudes toward air pollution, and by identifying the effects by country or segment.

## Materials and methods

### Survey overview

Online surveys were conducted to investigate the effects of information provision, implemented as a nudge, on recycling disposable plastics. Although we designed the experimental framework and the questionnaires, a survey company was hired to distribute the questionnaires and collect the responses. Respondents were registered users of the survey company living in Japan, Canada, and the US who were aged 20 years or older. The legal definitions of an adult were different across the three countries; adults were defined as 20 years or older in Japan at the time of the survey, whereas they were defined as 18 years or older in Canada and the US. To exclude any minors in all the three countries using the same age condition, we collected responses from those who were 20 years or older in the present survey. The company obtained written informed consent from all the participants on our behalf. The survey was anonymized and did not collect any personal information. No biological samples were obtained from the respondents, and they were assumed not to be subjected to any psychological distress as a result of their participation. The surveys were approved by all the relevant ethics committees of the Central Research Institute of Electric Power Industry in Japan and Saint Mary’s University in Canada. For the State University of New York at New Paltz’s contributions, data collection was not conducted as part of the work of that particular team. As such, their contributions were deemed as “non-human subjects research” in line with the policies of the university’s Human Research Ethics Board. This study was conducted in accordance with the Declaration of Helsinki and its later amendments.

**Fig 1:**
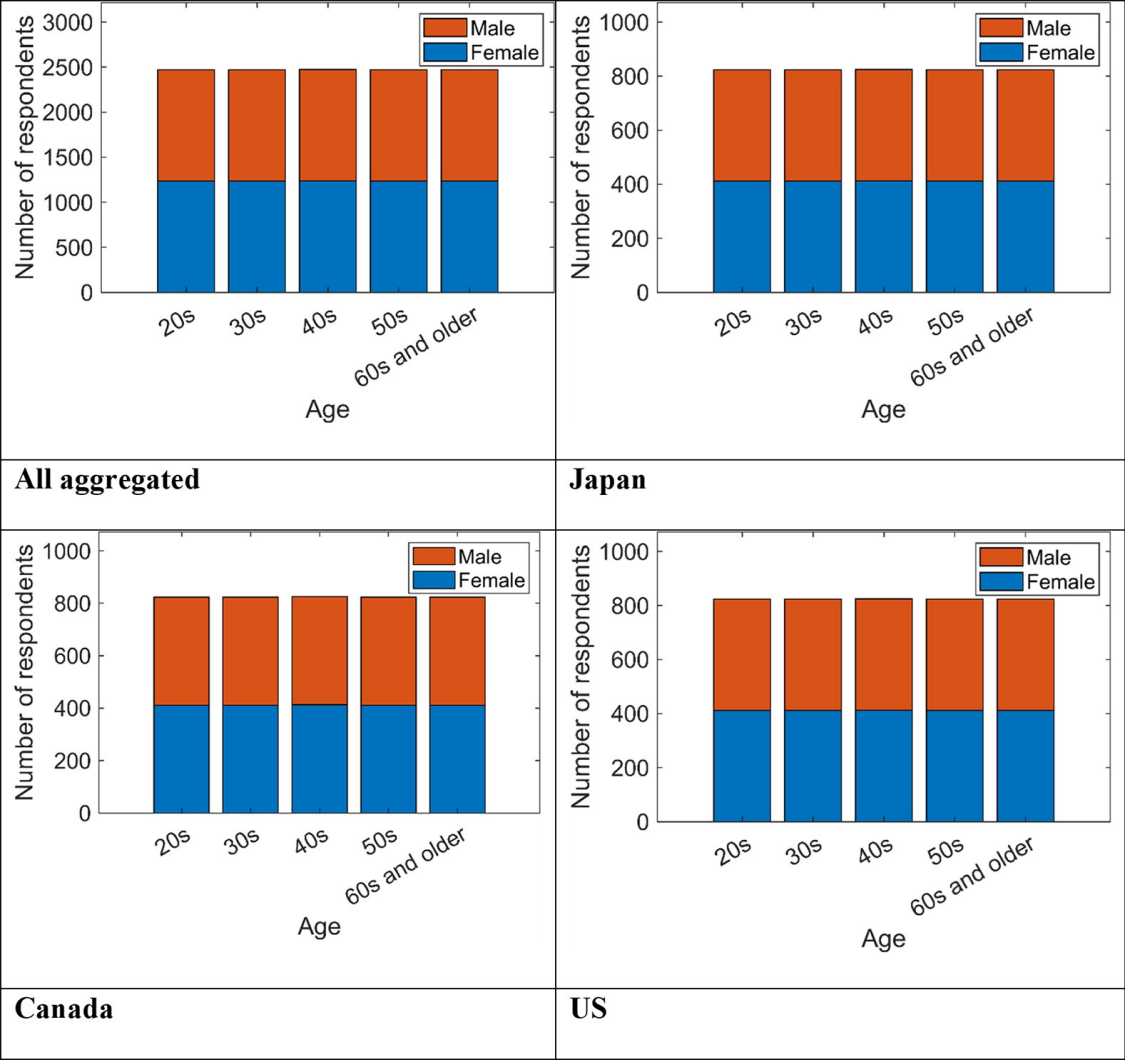
Number of respondents by age and sex.

We conducted the survey from February 10 to March 5, 2021 in the three countries (Table 1). Basic attributes of respondents, such as age and sex, were based on the survey company’s records. We sampled the responses so that the numbers of respondents were equal for each sex and each of the five age bins, and 10 segments comprising the same numbers of responses were obtained. The age bins were set to 20s, 30s, 40s, 50s, and 60s and older (Fig 1). The other attributes were based on incidence rate. We prepared the questionnaires in Japanese, English, and French. The respondents in Japan received the Japanese version and the respondents in the US received the English version. Although the respondents in Canada could choose the English or French version, all the respondents chose the English version. Table 1 shows the total number of valid samples and sex ratios for each country.

**Table 1:** Basic attributes of the three datasets.

Dataset	Japan	Canada	US
Survey term	Feb. 10–Feb. 16, 2021	Feb. 19–Mar. 5, 2021	Feb. 17–Mar. 1, 2021
Number of samples	4120	4120	4120
Sex ratio (%)	100.1	100.1	100.1

Table 2 shows the number of children, children living with the respondents, children working, parents living together, and parents working. We sampled the same question in our previous survey for another type of information provision in 2020 (S2 Appendix in S1 File). Despite the COVID-19 pandemic that occurred while we performed the present survey, these numbers for each country did not change greatly during the year in which the pandemic was also happening. The trend was almost constant and the three types of children were the largest in the US. The number of parents who were living together and working in paid jobs was the largest in Japan, although the differences from the other two countries were small.

**Table 2:** Characteristics of respondents’ children and parents.

Dataset	Japan	Canada	US
**Mean number of children**	1.1	1.1	1.4
**Mean number of children living with respondent**	0.6	0.6	0.8
**Mean number of children who are working**	0.5	0.5	0.6
**Mean number of parents living in the same house or at the same site**	0.4	0.3	0.3
**Mean number of parents working in paid jobs**	0.5	0.4	0.4

### Survey design

**Fig 2:**
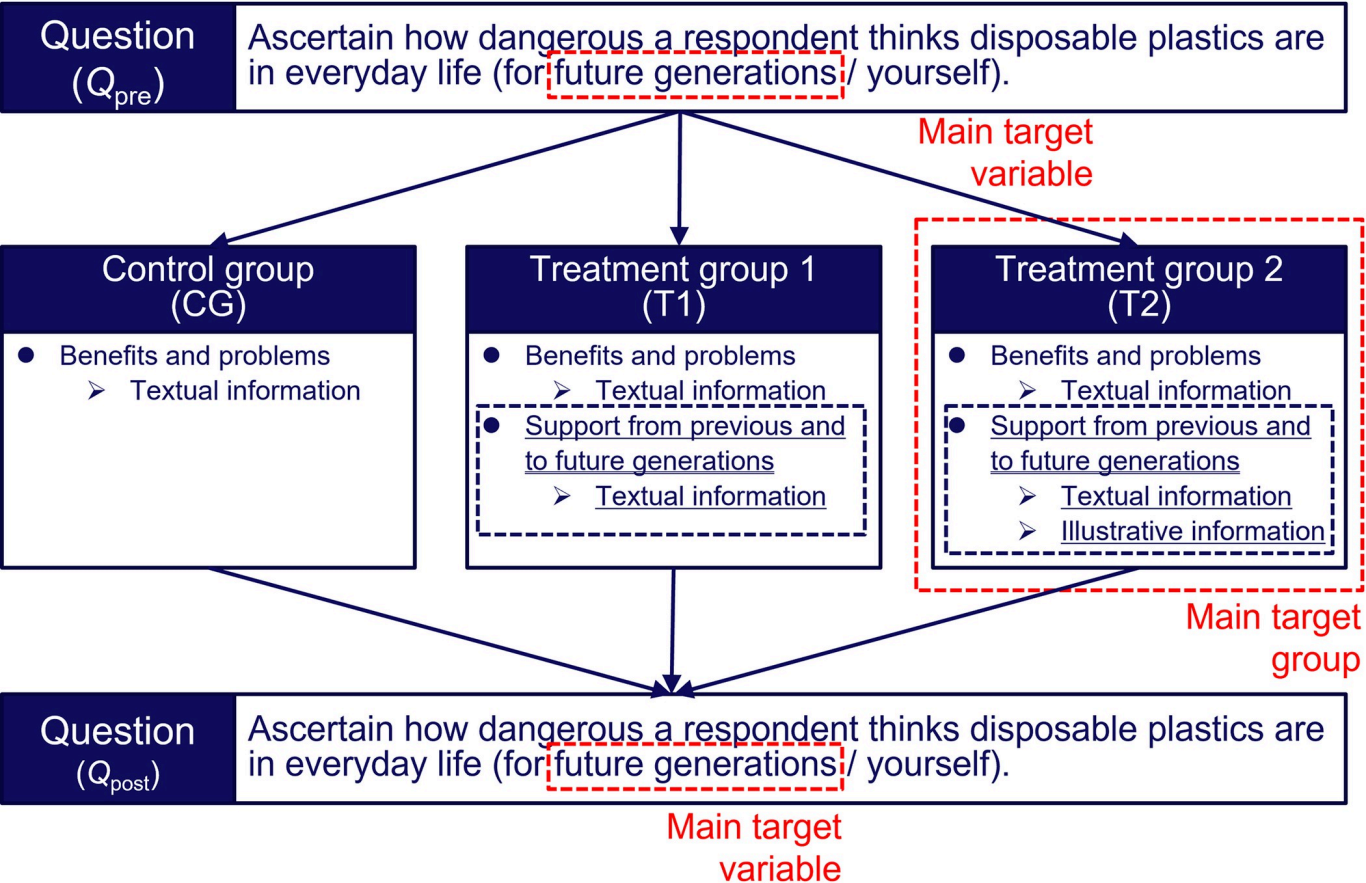
Experimental procedure for investigating nudging messages for recycling disposable plastics. The black dashed boxes show the interventions. The targets of the interventions are highlighted with red dashed boxes.

The present survey design followed our previous experimental frameworks [27] (S2 Appendix in S1 File) as shown in Fig 2. We performed a randomized controlled trial using online surveys to investigate the nudging message effects on moderating negative attitudes toward disposable plastics.

**Fig 3:**
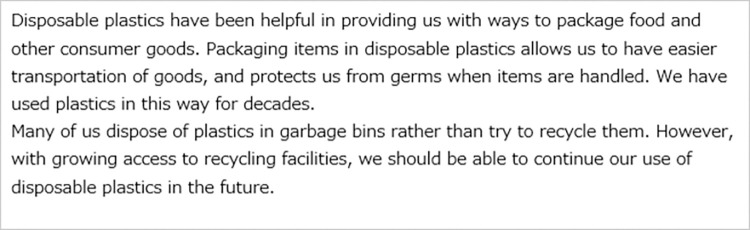
Message presented to the control group.

**Fig 4:**
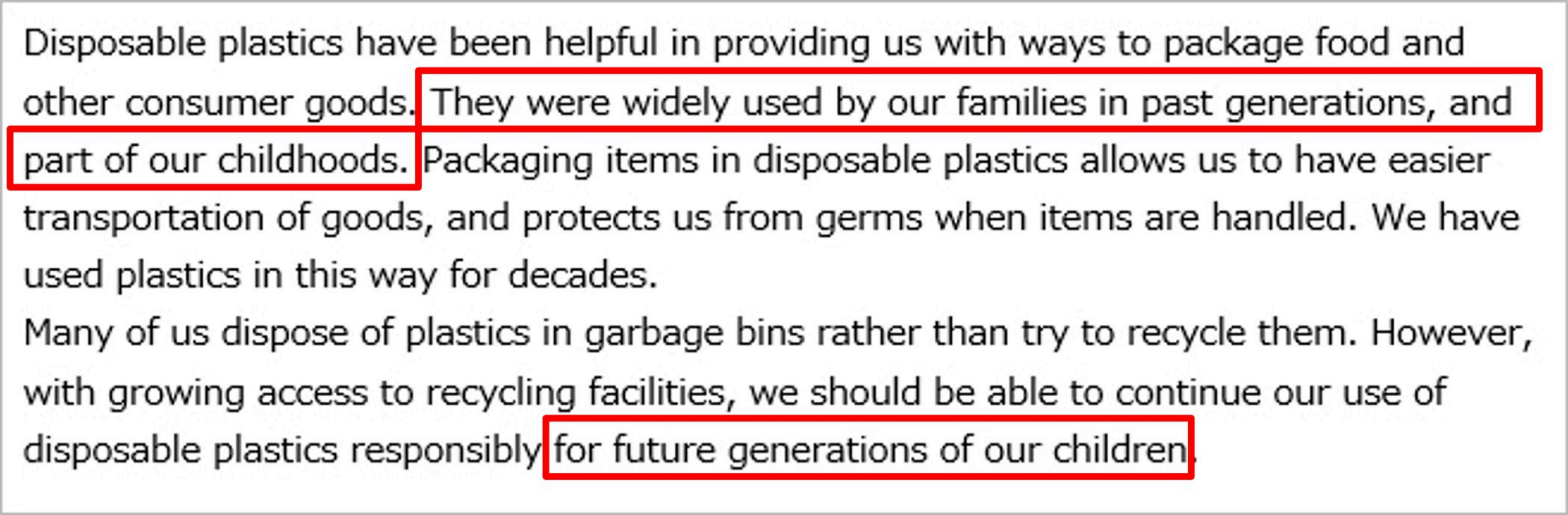
Message presented to treatment group 1. The intervention is highlighted with red rectangles.

**Fig 5:**
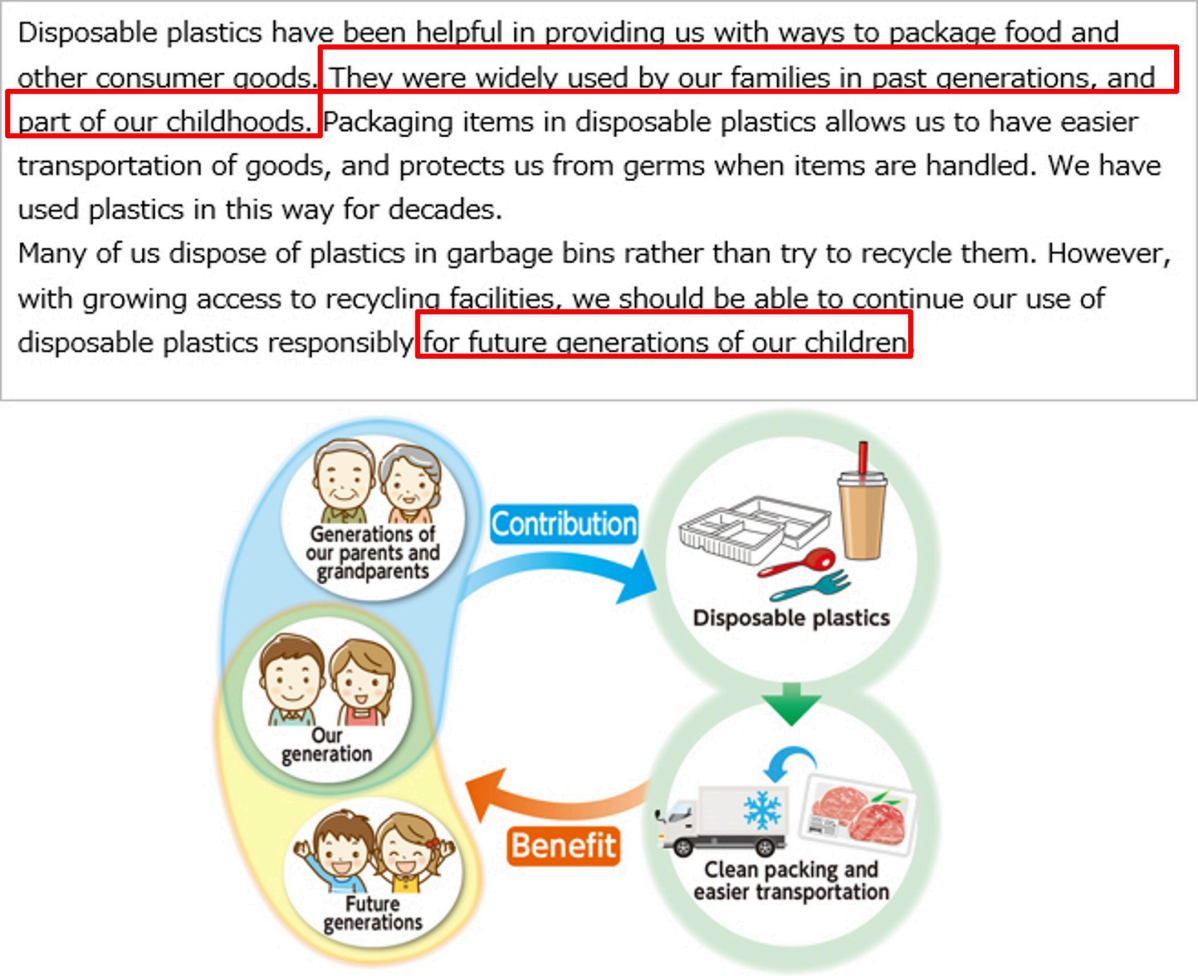
Message presented to those under 50 years of age in treatment group 2. The intervention is highlighted with red rectangles.

**Fig 6:**
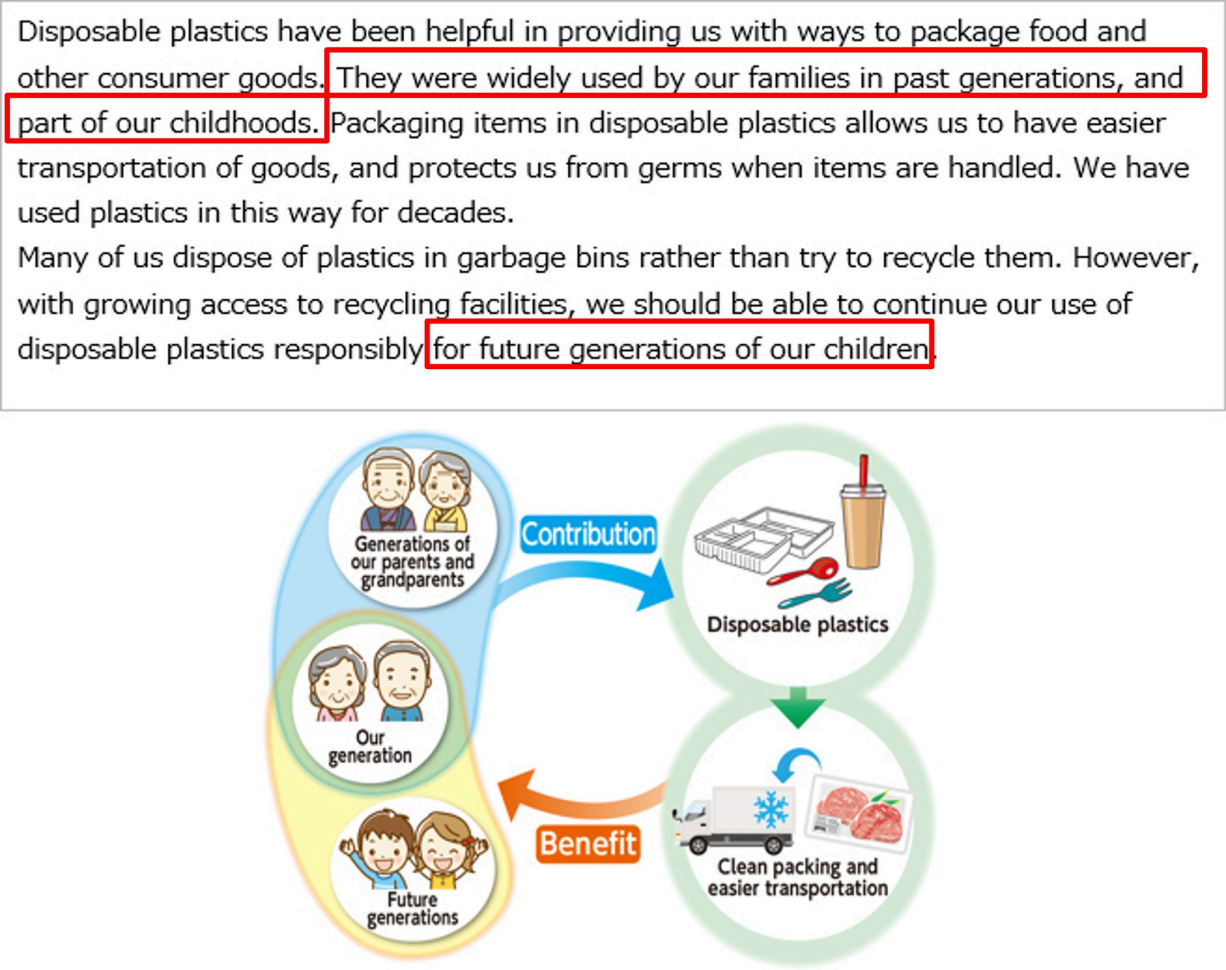
Message presented to those 50 years of age and older in treatment group 2. The intervention is highlighted with red rectangles.

All the respondents were first asked about the perceived risks of disposable plastics to Future Generations and Yourself in everyday life. These pre-intervention attitudes toward disposable plastics were defined as *Q*_*pre*_. Then the respondents were randomly assigned to one of the three message groups, control group (CG), treatment group 1 (T1), and treatment group 2 (T2). CG received the most basic textual information describing the benefits and problems with disposable plastics (Fig 3). T1 received textual information describing how previous generations are benefiting the respondents themselves and future generations via disposable plastics in addition to the information for CG (Fig 4). Our main target group T2 received illustrative information to highlight its textual contents in addition to the information for T1. The appearance of the characters in the left side of the illustrations was changed to match the respondents’ age (Figs 5 and 6). Table 3 summarizes the structure of the information presented to each message group.

**Table 3:** Summary of interventions.

Information provided	Presentation format	CG	T1	T2 (main target group)
Benefits and problems with disposable plastics	Text	✔	✔	✔
Support from previous generations/to future generations	Text		✔	✔
	Illustration			✔
**Number of samples**	Japan	1373	1374	1373
	Canada	1373	1374	1373
	US	1373	1374	1373

CG, control group; T1, treatment group 1; T2, treatment group 2. The check mark (✔) indicates that the information was provided.

CG received texts about the benefits and waste-related problems with disposable plastics so that the impression of the information was as neutral as possible. The benefits referred to using packaging to protect contents, such as food, to keep them clean and enable easy transportation. The waste-related problems highlighted were that many people still dispose of plastics and do not recycle them, despite recent advancements and better access to recycling facilities.

T1 received two additional sentences based on the most basic texts for the CG. One of the sentences mentioned that disposable plastics have a history and have been used in the previous generation, as well as by the current generation, suggesting that they have already been benefiting us for decades. The other additional sentence highlighted the support to future generations via the use of disposable plastics. The two additional sentences were designed to increase perceived support from older generations.

T2 received the same textual information (i.e., identical to T1), along with the additional illustration. The illustration visualized the relationship of how the older generations and one’s own generation were benefiting the current generation and future generations via disposable plastics. Owing to the wide range of participant age groups, the illustrations of the participants’ own generation and previous generations were changed according to age group (under 50 years or 50 years and older). Because those over 50 years old were less likely to have living parents, we presented older people in the traditional clothing that their parents and grandparents wore. This illustrative framework was based on our previous survey of nudges for increasing positive attitudes toward air pollution caused by industrialization [27] (S2 Appendix in S1 File).

The respondents were again asked the same question as for *Q*_pre_ before the intervention (*Q*_post_). Both of the questions ascertained the perceived risks of disposable plastics on Future Generations and Yourself before and after receiving the designed messages.

### Statistical analysis

The statistical analyses comprised three parts. The first part was analysis based on descriptive statistics for the initial status of respondents, such as the attitudes toward plastic recycling and COVID-19. The second part was a difference-in-difference (DID) estimation to identify the intervention effects by comparing a control group with treatment groups on how the attitude change differed across the groups. Then, correlation analyses were performed to investigate how perceived support by older generations via recycling disposable plastics and perceived support of future generations that benefit from the recycling were correlated with the message effects. Estimation of the message effects based on descriptive statistics was also conducted by segment, such as sex and age. The third part was a panel analysis based on forced-entry linear regression models to extract the intervention effects more precisely by separating the influence of the respondents’ basic attributes and personality traits.

To identify our designed message effects in all the analyses, we set two separate explained variables, which were perceived risks of disposable plastics to future generations (Future Generations) and those to the respondents themselves (Yourself). Although we evaluated both of the explained variables in parallel, our main target variable was Future Generations to determine the effectiveness of the nudging message.

Statistical significance of the differences across our segments was calculated using the Wilcoxon signed-rank test. All error bars in graphs were computed as 95% confidence intervals based on *t*-distributions. All of these analyses were performed using Matlab R2021b with the Statistical and Machine Learning Toolbox.

## Results

### Before interventions

**Fig 7:**
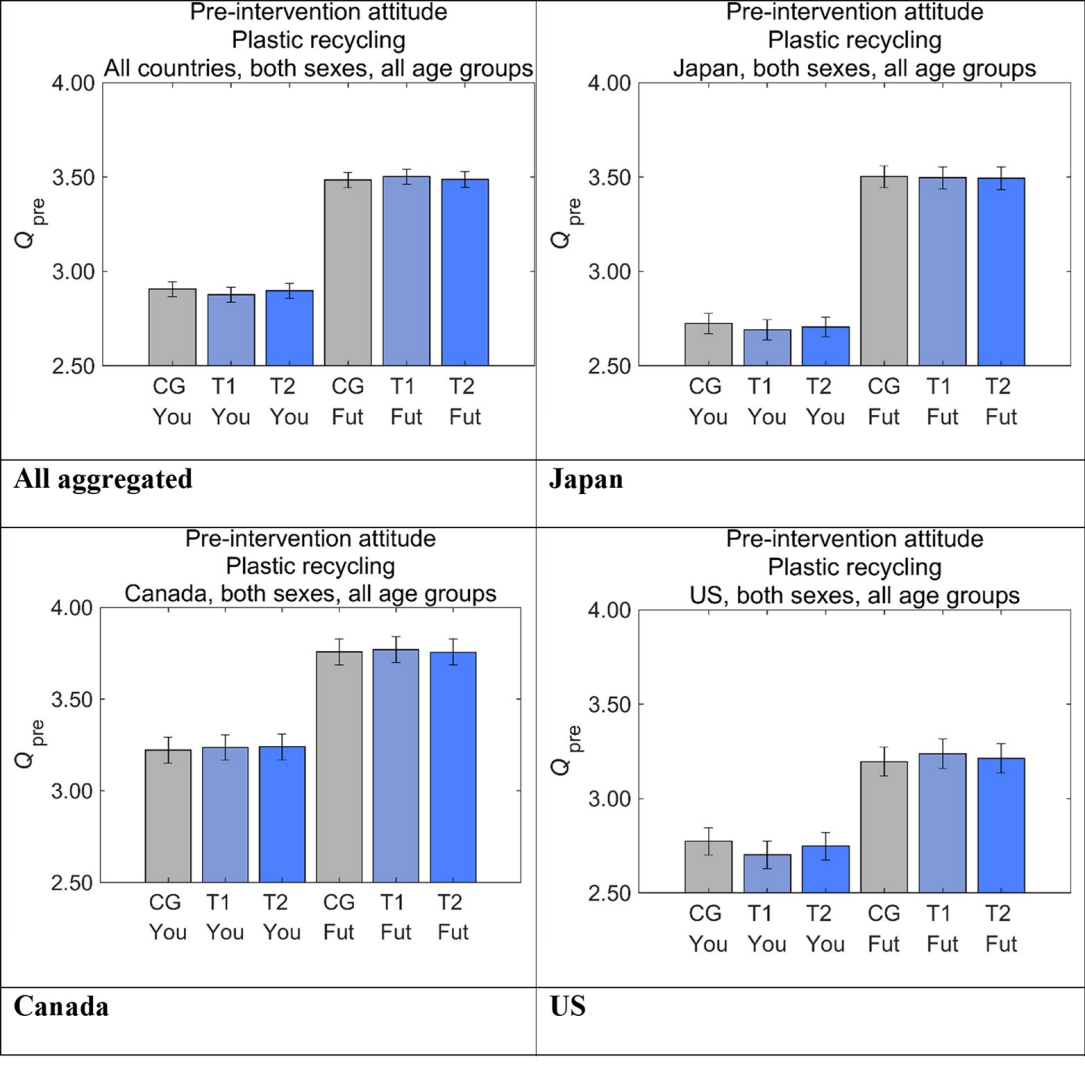
Risk perception on disposable plastics by group before receiving the designed messages. Higher values on the vertical axis indicate higher perceived risks. Error bars show 95% confidence intervals. CG, control group; T1, treatment group 1; T2, treatment group 2; Fut, Future Generations; You, Yourself.

Fig 7 shows the pre-intervention attitudes toward disposable plastics (*Q*_pre_) on Future Generations and Yourself, where ‘All aggregated’ provides the aggregated results of the three countries. We use the same format for all figures to compare the results across the countries.

In all of the countries, there was a strong tendency for *Q*_pre_ to be larger for Future Generations than for Yourself, suggesting that disposable plastics are considered more dangerous to Future Generations than to Yourself. However, there were also some differences across the countries. Both *Q*_pre_ for Future Generations and Yourself were the largest in Canada, and *Q*_pre_ for Future Generations in Japan was larger than the US and showed the second largest difference. *Q*_pre_ for Yourself in Japan and the US were the smallest and were similar. These results suggested that Canadians may be most concerned about disposable plastics, whereas Americans may be least concerned.

Within Future Generations or Yourself for the same country, there were no significant differences among the three message groups, suggesting that the samples were well randomized.

Focusing on our main target variable, Future Generations, the *Q*_pre_ values were consistently larger than 3 in all the three countries. Considering that 3 was ‘neutral’ and 5 was ‘dangerous’ on the 5-point Likert scale, perceptions of the respondents were slightly biased to ‘dangerous,’ showing the risk-averse attitudes toward disposable plastics.

### Attitudes toward COVID-19

The COVID-19 pandemic has had a wide variety of effects on lifestyles [33, 34] and perceptions of risk have changed [35], causing unexpected problems, such as social isolation [36] and damage to mental health [37]. Our survey was conducted in the three countries during the COVID-19 pandemic, which may have also altered the effects of our designed message. Thus, we ascertained the cultural differences in risk perception of COVID-19 as background data.

**Fig 8:**
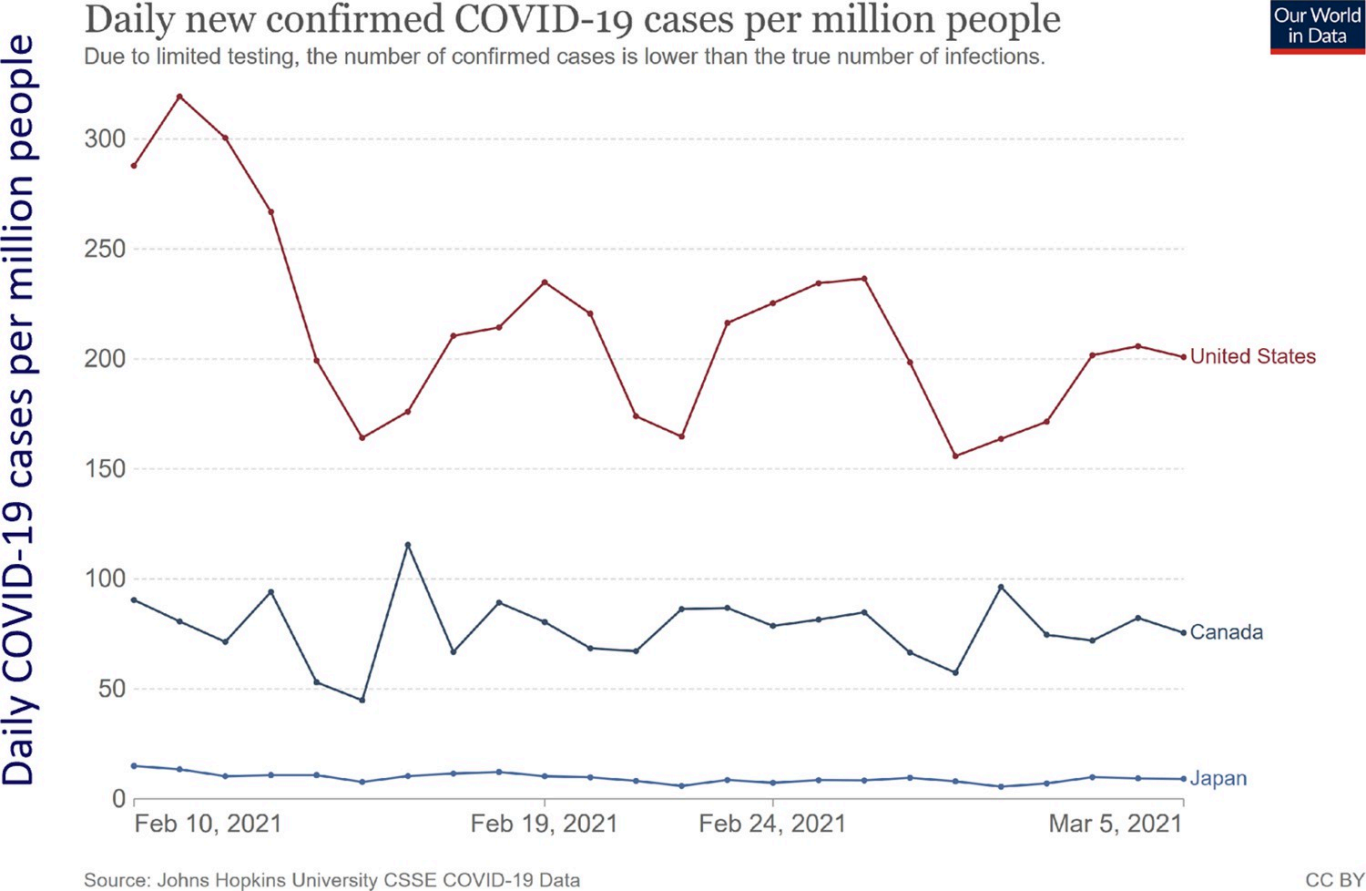
Daily COVID-19 cases per million people by country from February 10 to March 5, 2021. Retrieved from [38], the dataset of which is based on the COVID-19 Data Repository of the Center for Systems Science and Engineering at Johns Hopkins University.

**Fig 9:**
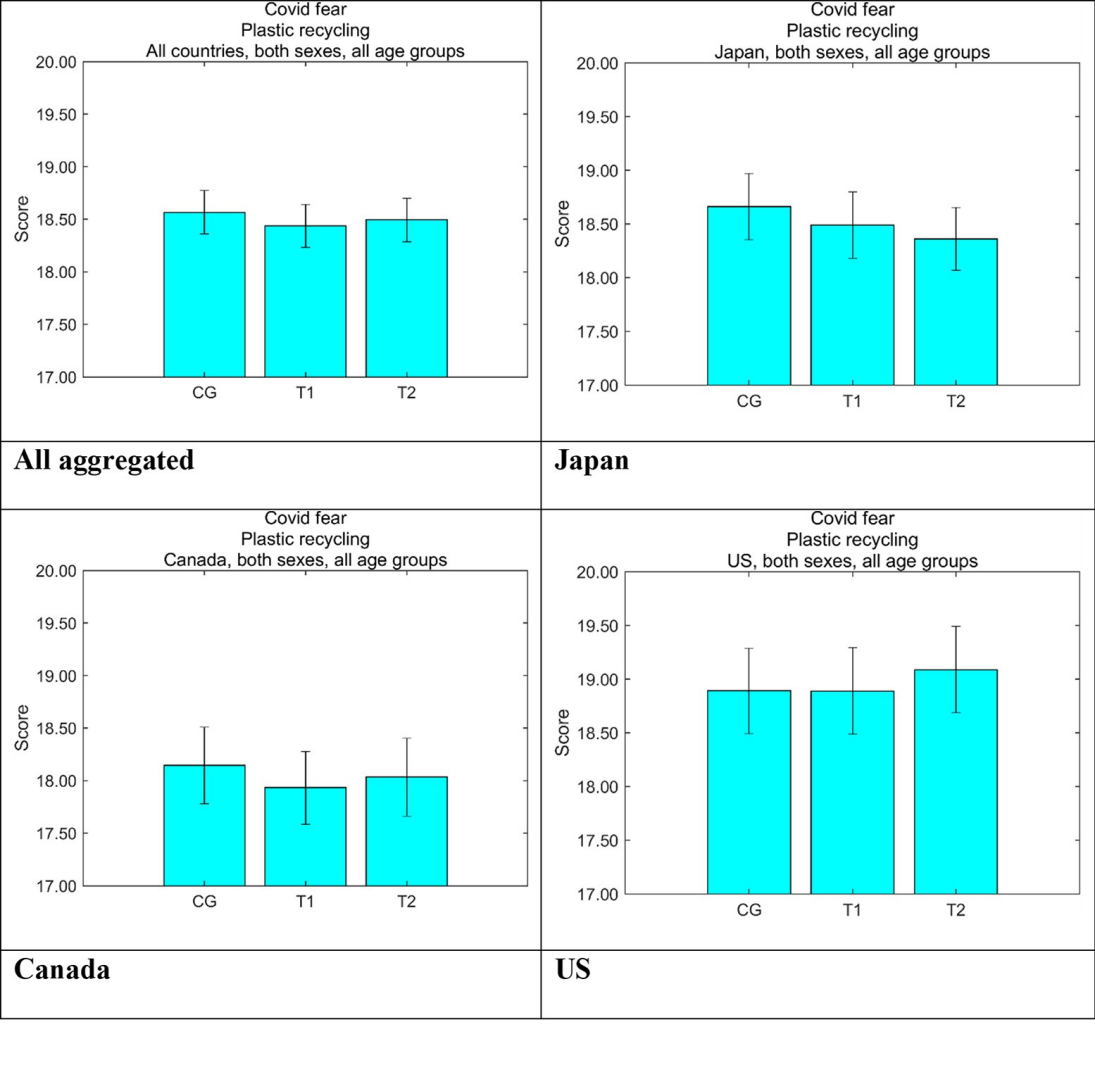
Fear about COVID-19 by group. Higher values on the vertical axis indicate higher perceived danger of COVID-19. Error bars show 95% confidence intervals. CG, control group; T1, treatment group 1; T2, treatment group 2.

Fig 8 shows the daily COVID-19 cases per million by country [38, 39] during the survey term. The number of the cases were in the order US > Canada > Japan, and the differences were clear. Fig 9 shows the perceived personal risks in each country by message group, which was measured by established scales [40]. The scale comprises seven questions on a five-point Likert scale with a median score of 21. Although there were differences among the three countries, all the average scores were lower than the median, suggesting that the risk perceptions were low. The US showed the highest risk perception corresponding to the reality of the pandemic, Japan showed a higher risk perception for COVID-19 than Canada, which showed the lowest risk perception, although the actual cases in Japan were by far the lowest in the three countries during this period. Cronbach’s α calculated by using all the responses from all the three countries was 0.901, which suggested that the internal reliability for the scales was sufficiently high.

**Fig 10:**
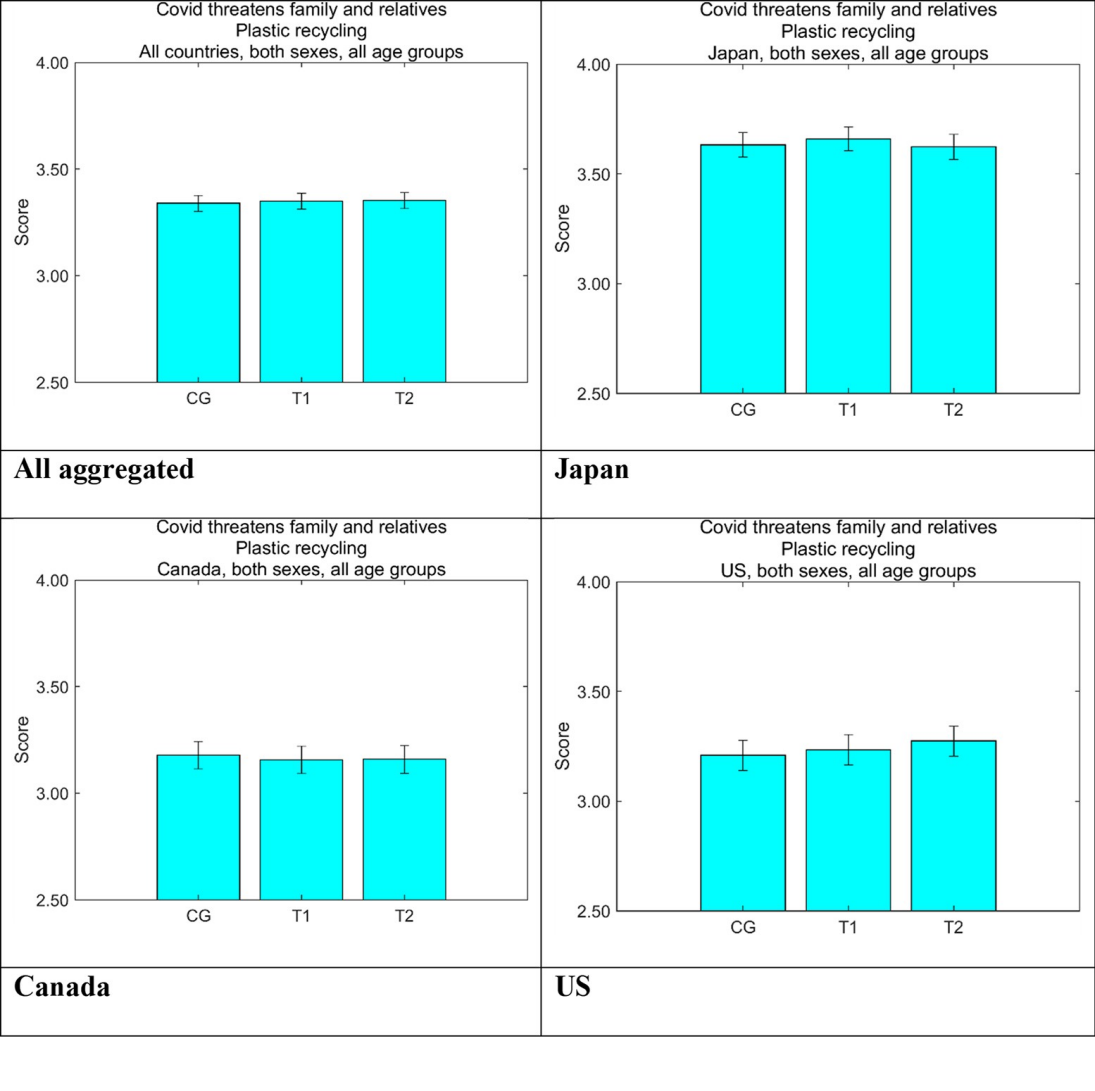
Perception by group that COVID-19 threatens the respondents’ family and relatives. Higher values on the vertical axis indicate higher perceived danger. Error bars show 95% confidence intervals. CG, control group; T1, treatment group 1; T2, treatment group 2.

Considering that our nudging messages were designed to promote a sense of familial support, we ascertained people’s perceptions of how much COVID-19 threatened family and relatives (Fig 10). The median score was 3 because we used a five-point Likert scale for this question. All three countries scored more than the median. Japan showed by far the highest scores, whereas the other two countries showed similar scores although the reality was that the number of cases was lowest in Japan. Although the scale for COVID-19-related fear [40] focuses on the personal risk perception, respondents in Japan might be more concerned about their family, rather than their own health.

### Overall intervention effects

**Fig 11:**
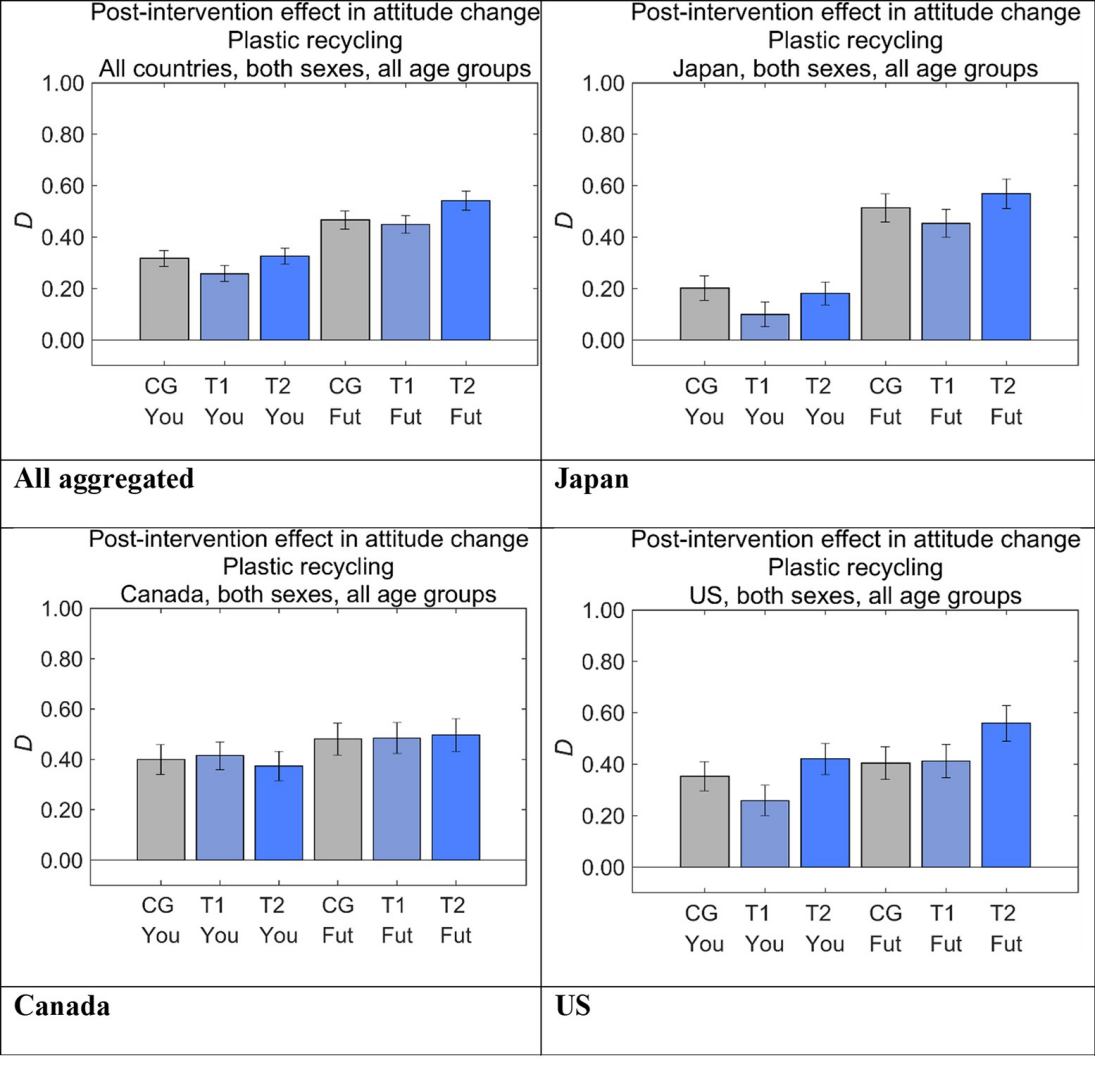
Post-intervention effect in attitude change toward disposable plastics (*D*). Higher values on the vertical axis indicate lower perceived danger of disposable plastics. Error bars show 95% confidence intervals. CG, control group; T1, treatment group 1; T2, treatment group 2; Fut, Future Generations; You, Yourself.

We defined the degree of post-intervention in attitude change toward disposable plastics as *D*, which was the difference between the answers to *Q*_post_ and *Q*_pre_ in each sample. Fig 11 shows *D* for each country and the aggregated results by message group, where the effects are shown for Future Generations and Yourself. All the message effects for all the countries were positive and the effect sizes were consistently larger for Future Generations than for Yourself. Although there were differences in effect sizes among the message groups within Future Generations or Yourself in the US and Japan, there were no significant differences among the three message groups in Canada for both Future Generations and Yourself. Although this homogeneity of effect was not self-evident, it may have been influenced by the governmental declaration that Canada would ban use of disposable plastics by the end of 2021 [5], which was enacted as a regulation later [41]. One possible interpretation is that there is no choice in using disposable plastics, and hence the message contents do not matter for people in Canada if disposable plastics are going to be officially banned. In contrast, there are no country-wide regulations in the US and Japan banning disposable plastics, although a small number of states in the US have been restricting them.

Comparing the same message groups in the same country, Future Generations showed consistently higher scores than Yourself for *D*. Target group T2 for Future Generations had a significantly larger score than CG (i.e., positive) in the aggregated results (*p* < 0.01), which was mainly caused by the largest difference between groups in the US and the second largest difference in Japan. However, the difference between the scores for T2 and CG for Future Generations was not significant. The score for T1 for Yourself was significantly lower than that for CG (i.e., negative) in the aggregated results (*p* < 0.001), which was mainly caused by the largest difference between scores in Japan and the second largest difference in the US. The difference between the scores for T1 and CG for Yourself was not significant.

These results suggest that the T2 message should be used instead of the T1 message to increase people’s positive attitudes toward disposable plastics, even if the content of the two messages is essentially the same. The only difference between the T2 and T1 messages was the additional illustration showing who is supporting whom via disposable plastics. Thus, the effects of the illustration canceled the negative effect of T1 and even increased the message effect, as seen in the larger effect in T2 compared with CG for Future Generations.

**Fig 12:**
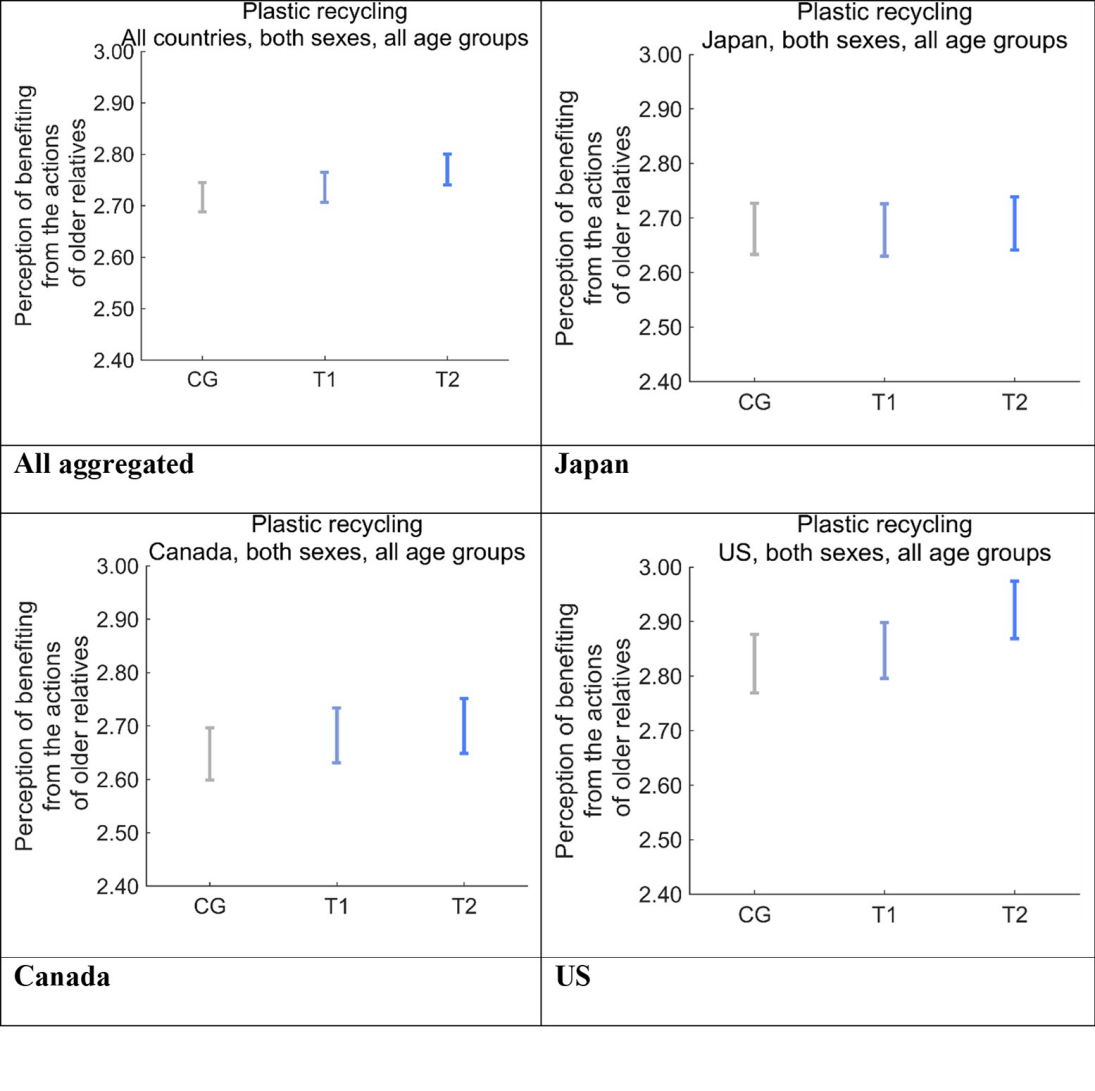
Perception that the respondent has benefited from the actions of relatives belonging to older generations after receiving one of the nudge message interventions. Error bars show 95% confidence intervals. CG, control group; T1, treatment group 1; T2, treatment group 2.

We ascertained the degree to which respondents feel their health and quality of life are supported by older generations, including their parents and grandparents (Fig 12). The perceived benefits were the largest in the US on average. This comparison yielded the finding of highest to lowest perceived support scores of T2 > T1 > CG in the US and Canada, with differences clearer in the US than in Canada, which resulted in the same order of aggregated perceived support scores. The perceived support scores for the three message groups were the same in Japan. The significant differences were T2 > CG in the US (*p* < 0.01) and T2 > CG in Canada (*p* < 0.1).

**Fig 13:**
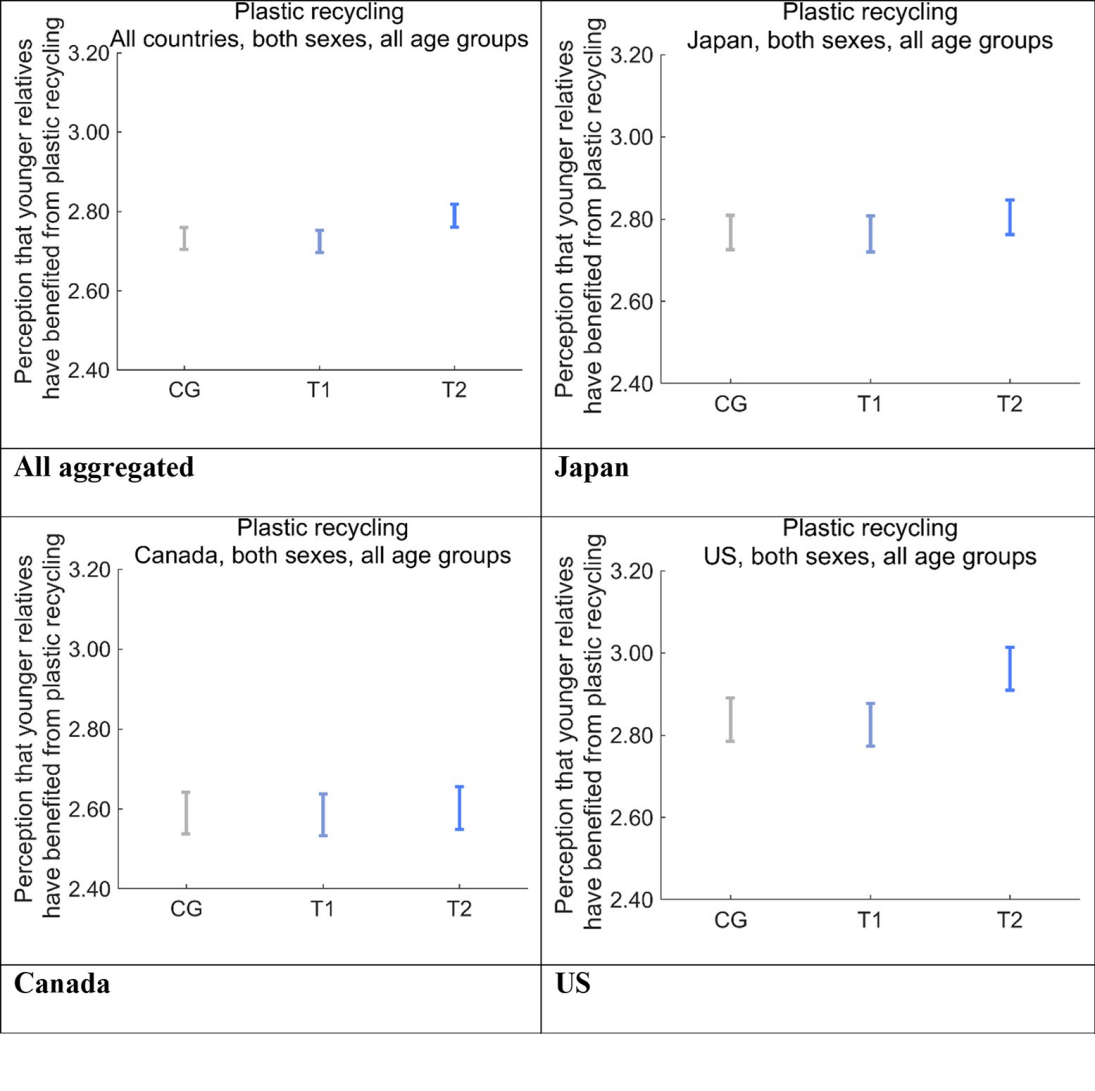
Perception that the respondents’ younger relatives have benefited from disposable plastics after receiving one of the nudge message interventions. Error bars show 95% confidence intervals. CG, control group; T1, treatment group 1; T2, treatment group 2.

Furthermore, we ascertained the degree to which the respondents feel the health and quality of life of their younger relatives, including their children and grandchildren, are supported by disposable plastics (Fig 13). The scores for perceived support to younger relatives was in the order US > Japan > Canada, on average. The scores for CG and T1 were similar in all three countries, whereas that for T2 was larger than those for CG and T1 in the US and Japan, and the difference was significant only in the US (*p* < 0.001). The aggregated results showed that the score for T2 was significantly larger than that for T1 (*p* < 0.01) and CG (*p* < 0.001).

The differences between the scores for T2 and CG were the largest for both perceived support from older generations (Fig 12) and perceived support to younger generations in the US (Fig 13), which had the largest DID effect for Future Generations (Fig 11).

We investigated how our designed messages were correlated with the perceived support from the older generations (Table 4) and with the perceived support to younger relatives via disposable plastics (Table 5). For most of the combinations of dataset and message group, the statistical significance was strong (*p* < 0.001). The correlation coefficients were in the order Future Generations > Yourself and T2 > T1 > CG for the aggregated datasets. This finding suggests that our designed nudging messages increased the sense of familial support and the DID effect, especially for our target group T2 for Future Generations.

**Table 4:** Correlation coefficients between post-intervention effect in attitude change (*D*) and perceptions of benefiting from the older generations.

Dataset	All aggregated		Japan		Canada		US	
Group	Future Generations	Yourself	Future Generations	Yourself	Future Generations	Yourself	Future Generations	Yourself
**All aggregated**	0.102***	0.087***	0.120***	0.080***	0.101***	0.106***	0.095***	0.070***
**CG**	0.078***	0.069***	0.125***	0.110***	0.057*	0.062*	0.070**	0.046^†^
**T1**	0.090***	0.085***	0.102***	0.086**	0.100***	0.110***	0.076**	0.064*
**T2**	0.135***	0.105***	0.132***	0.045^†^	0.142***	0.146***	0.130***	0.093***

CG, control group; T1, treatment group 1; T2, treatment group 2.

^†^, *, **, *** Difference from zero with 90%, 95%, 99%, and 99.9% confidence, respectively.

**Table 5:** Correlation coefficients between post-intervention effect in attitude change (*D*) and perceptions that younger relatives are benefiting rom disposable plastics.

Dataset	All aggregated		Japan		Canada		US	
Group	Future Generations	Yourself	Future Generations	Yourself	Future Generations	Yourself	Future Generations	Yourself
**All aggregated**	0.181***	0.144***	0.186***	0.130***	0.206***	0.185***	0.162***	0.129***
**CG**	0.159***	0.129***	0.189***	0.137***	0.173***	0.167***	0.133***	0.100***
**T1**	0.182***	0.142***	0.156***	0.125***	0.222***	0.197***	0.172***	0.133***
**T2**	0.200***	0.158***	0.209***	0.129***	0.221***	0.191***	0.171***	0.147***

CG, control group; T1, treatment group 1; T2, treatment group 2.

^†^, *, **, *** Difference from zero with 90%, 95%, 99%, and 99.9% confidence, respectively.

In the previous study for air pollution caused by industrialization, these correlation coefficients were similar for both the perceived support from older generations and the perceived support to younger relatives (Tables A and B in S2 Appendix in S1 File). However, in the present study, the coefficients for the perceived support to younger relatives were larger than those for the perceived support from older generations. One possible interpretation for this difference is that the respondents thought it would be great for future generations if both recycling and using disposable plastics could go together. Disposable plastic is a hot topic that attracts much attention in the context of SDGs, where contributions for future generations are emphasized, which could potentially promote such future-oriented attitudes. In contrast, air pollution caused by industrialization has been greatly improved and is a less important problem long after high-growth periods. Thus, air pollution might show less attitudinal movement in terms of how it might benefit future generations (via the DID effects), compared with the timely topic of disposable plastics.

### Intervention effects by segment

**Fig 14:**
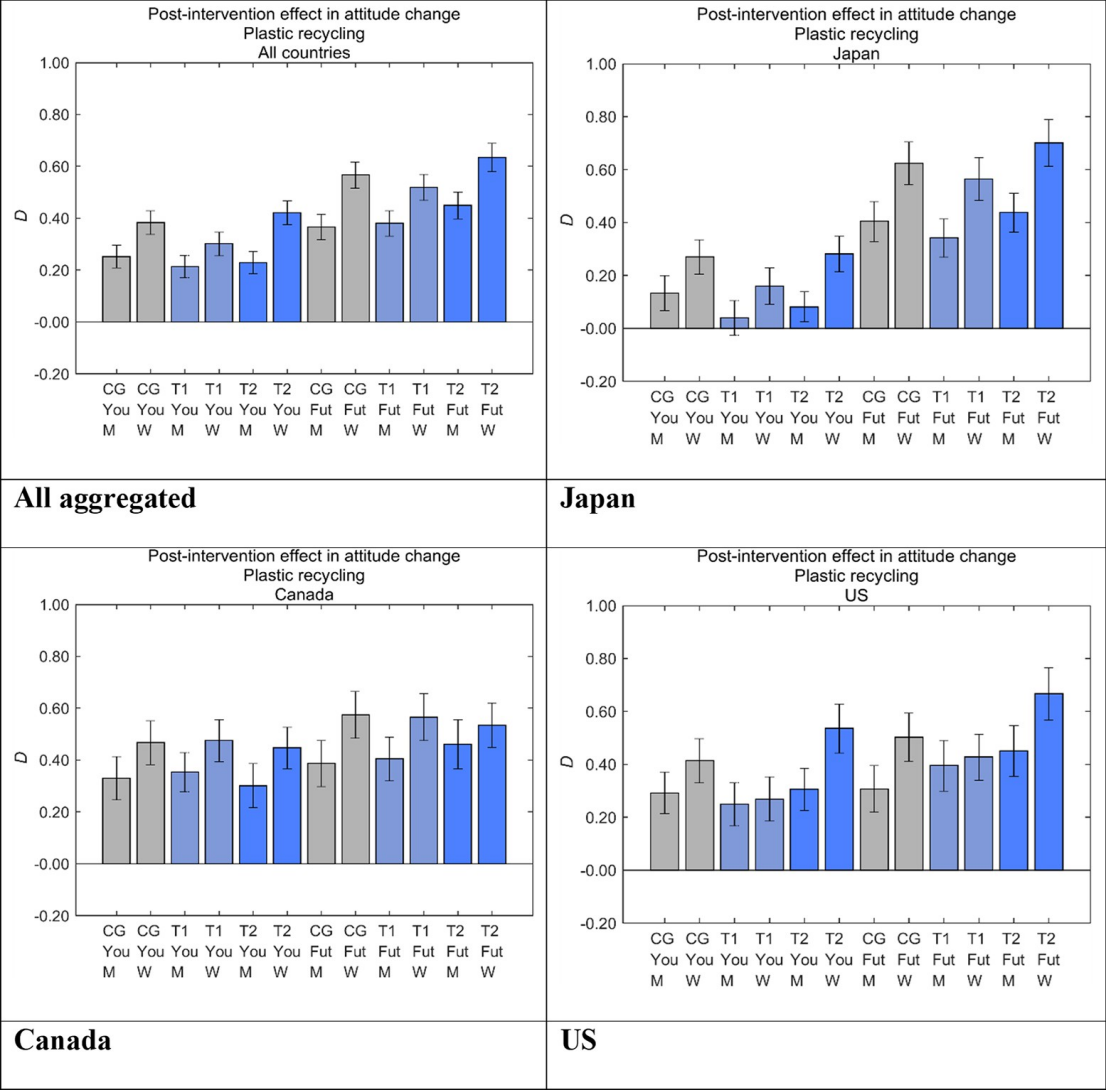
Post-intervention effect in attitude change toward disposable plastics (*D*) by sex. Error bars show 95% confidence intervals. Higher values on the vertical axis indicate lower perceived danger of plastic recycling. CG, control group; T1, treatment group 1; T2, treatment group 2; Fut, Future Generations; You, Yourself; M, men; W, women.

To investigate differences resulting from our designed messages by sex, we divided the samples in each message group into men and women (Fig 14). In all the message groups and countries, women showed higher *D* than men. Although the statistical significance of the differences between the message groups was sometimes unclear at the country level, the aggregated results showed significant differences for all the message groups (*p* < 0.001), with the only exception of T1 for Yourself (*p* < 0.05).

In the previous study for air pollution caused by industrialization, the sex differences in *D* were not observed in Canada (S2 Appendix in S1 File), whereas the present study showed clear sex differences. Thus, our designed messages, at least for disposable plastics, had a stronger effect for increasing positive attitudes of women than men in all three countries, different from our previous nudging messages for air pollution caused by industrialization [27] (S2 Appendix in S1 File).

**Fig 15:**
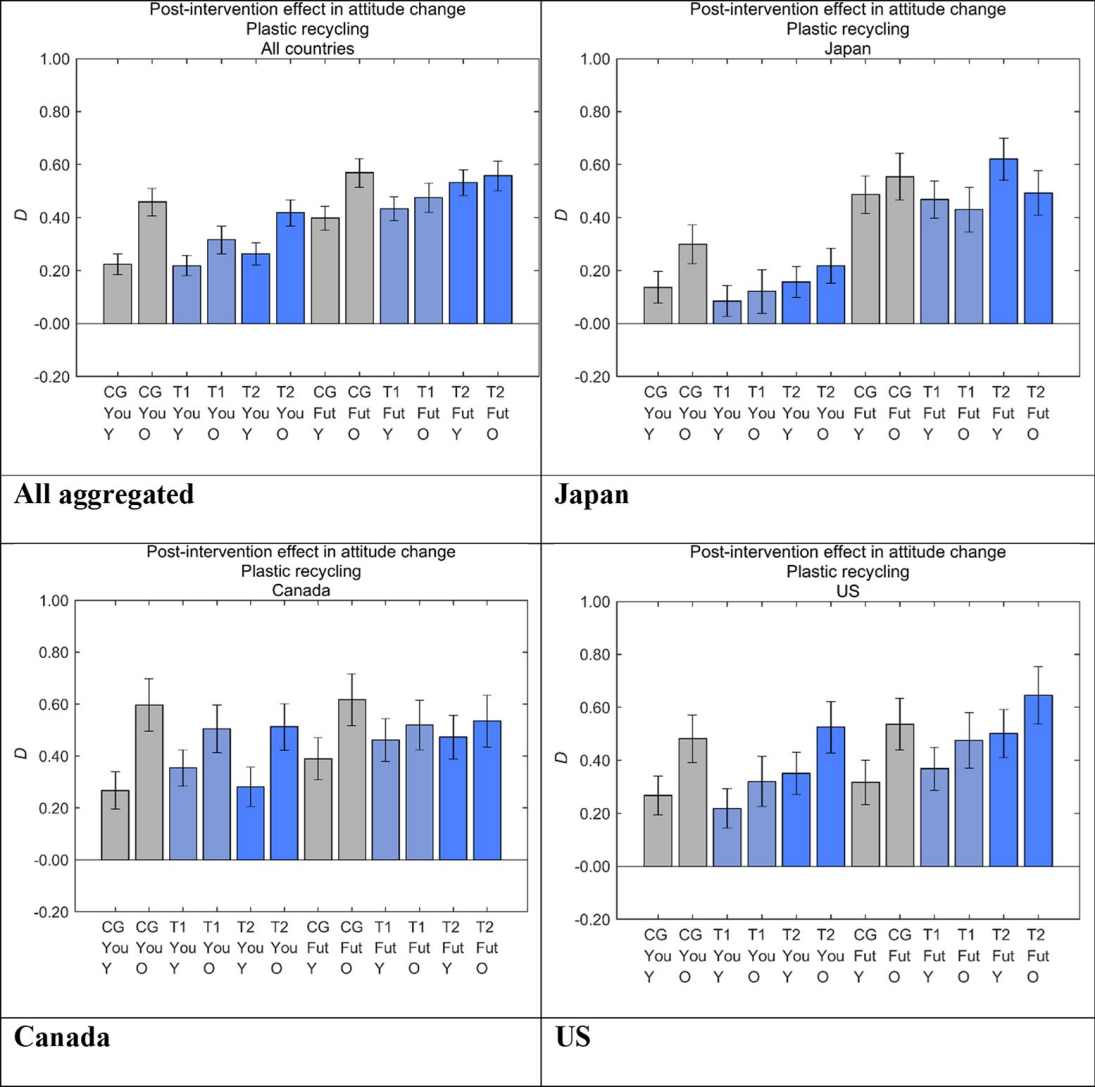
Post-intervention effect in attitude change toward disposable plastics (*D*) by age. Error bars show 95% confidence intervals. Higher values on the vertical axis indicate lower perceived danger of plastic recycling. CG, control group; T1, treatment group 1; T2, treatment group 2; Fut, Future Generations; You, Yourself; Y, respondents under the age of 50 years; O, respondents 50 years of age and older.

To investigate differences in our designed messages by age, we divided the samples in each message group into younger (under the age of 50) and older (50 years and older) respondents (Fig 15). Older respondents showed larger *D* than younger respondents in all message groups and countries, with the two exceptions of T1 and T2 for Future Generations in Japan. The aggregated results showed larger message effects for older respondents in all the message groups although the differences were not always significant. The differences were especially small in T2 for Future Generations, which is our main target for interventions, and in T1 for Future Generations. The trend in the previous study for air pollution caused by industrialization was that younger respondents showed larger *D* than older respondents [27] (S2 Appendix in S1 File), which was the opposite of that in the present study.

### Panel data analysis

We constructed a linear regression model to identify the intervention effects of our nudging messages on increasing positive attitudes toward disposable plastics. The model consisted of dummies representing message groups T1 and T2, countries Canada and the US, and sex. Age was discretized into five classes of 20s, 30s, 40s, 50s, and 60s and older. Personality traits were incorporated using the Big Five and the Dark Triad framework, which were measured using the Ten-item Personality Inventory [42, 43] and the Dark Triad Dirty Dozen [44, 45], respectively. The two COVID-related explanatory variables, *C*_*fear*_ and *C*_*family*_, were the degree of how much the respondents were scared of COVID-19 [40] (Fig 9) and the perception of how much COVID-19 was threatening their family and relatives (Fig 10), respectively.


$$D=a_{1}\times T1+a_{2}\times T2+a_{3}\times Canada+a_{4}\times US+a_{5}\times S+a_{6}\times A+a_{7}\times P_{ex}+a_{8}\times P_{ag}+a_{9}\times P_{co}+a_{10}\times P_{ne}+a_{11}\times P_{op}+a_{12}\times D_{ma}+a_{13}\times D_{ps}+a_{14}\times D_{na}+a_{15}\times C_{fear}+a_{16}\times C_{family}+a_{17}$$ (1)

*T*1, *T*2: Target of the intervention in T1 and T2, respectively (0: no, 1: yes)
*Canada*: Living in Canada (0: no, 1: yes)
*US*: Living in the US (0: no, 1: yes)
*S*: Sex (0: men; 1: women)
*A*: Age
*P*_*ex*_: Extraversion (Big Five)
*P*_*ag*_: Agreeableness (Big Five)
*P*_*co*_: Conscientiousness (Big Five)
*P*_*ne*_: Neuroticism (Big Five)
*P*_*op*_: Openness (Big Five)
*D*_*ma*_: Machiavellianism (Dark Triad)
*D*_*ps*_: Psychopathy (Dark Triad)
*D*_*na*_: Narcissism (Dark Triad)
*C*_*fear*_: Degree of how much the respondents are afraid of COVID-19
*C*_*family*_: Perception of COVID-19 threats to relatives and family
*a*_*1*_–*a*_*16*_: Coefficients for each term
*a*_*17*_: Intercept

*D* is the difference between the answers to *Q*_post_ and *Q*_pre_ for Future Generations or Yourself. The coefficients and the intercept were determined by a forced entry regression method by pooling all the datasets from the three countries (Table 6).

**Table 6:** Coefficients from linear regression analysis.

Explained variables			Future Generations			Yourself		
			Estimated coefficients	SE	*t*	Estimated coefficients	SE	*t*
**Intercept**			0.589***	0.130	4.532	-0.038	0.115	-0.332
**Intervention**	T1		-0.020	0.025	-0.805	-0.063**	0.022	-2.806
	T2		0.074**	0.025	2.906	0.006	0.022	0.259
**Country**	Canada		-0.055^†^	0.030	-1.872	0.187***	0.026	7.121
	US		-0.082**	0.029	-2.778	0.138***	0.026	5.281
**Attribute variables**	Sex (men = 0, women = 1)		0.107***	0.022	4.880	0.080***	0.019	4.097
	Age (20s, 30s, 40s, 50s, and 60s and older)		-0.006	0.008	-0.787	0.034***	0.007	4.769
**Personality variables**	Big Five	Extraversion	0.007^†^	0.004	1.732	0.007^†^	0.004	1.858
		Agreeableness	0.022***	0.006	3.948	0.016**	0.005	3.254
		Conscientiousness	0.008	0.005	1.639	0.010*	0.004	2.241
		Neuroticism	0.006	0.005	1.326	0.008*	0.004	2.009
		Openness	0.008^†^	0.005	1.713	0.011*	0.004	2.514
	Dark Triad	Machiavellianism	-0.012***	0.003	-3.713	-0.009**	0.003	-3.054
		Psychopathy	-0.011***	0.003	-3.560	-0.011***	0.003	-3.805
		Narcissism	0.006*	0.003	2.412	0.004^†^	0.002	1.801
**COVID-19**	Fear		-0.013***	0.002	-7.223	-0.008***	0.002	-4.788
	Influence on family		0.093***	0.010	9.172	0.057***	0.009	6.325
**Adjusted R-squared**			0.0371			0.0299		
**Number of valid samples**			12360			12360		

^†^, *, **, and *** indicate the difference from zero with 90%, 95%, 99%, and 99.9% confidence, respectively

*T*2 was estimated as 0.074 (*p* < 0.01) for Future Generations, suggesting that the intervention increased *D* in our target group compared with CG. *T*2 for Yourself was not significant, but importantly, it was estimated as not negative. *T*1 for Future Generations was estimated as negative, which was not significant, and *T*1 for Yourself was estimated as -0.063 and was significant (*p* < 0.01). These results suggest that the T2 message, which was the same message as T1 with additional illustrations, should be used to increase *D* instead of the T1 message, although the T2 message was not necessarily effective for Yourself.

For both *Canada* and *US*, the coefficients were estimated as negative for Future Generations and positive for Yourself, suggesting that the messages were more effective for Future Generations than for Yourself in Japan. *S* was estimated as 0.107 (*p* < 0.001) for Future Generations and 0.080 (*p* < 0.001) for Yourself, suggesting that women contributed more to *D* than men. This effect was caused by the stronger message effects for women, which were observed in most of the message groups of all the countries, as seen in Fig 14. *A* for Future Generations was not significant, whereas *A* was estimated as 0.034 (*p* < 0.001) for Yourself. This weaker age effect may be caused by the reversed effects in T1 and T2 in Japan (Fig 15). Agreeableness was the largest contributor of the Big Five traits, estimated as 0.022 (*p* < 0.001) and 0.016 (*p* < 0.01) for Future Generations and Yourself, respectively. This positive contribution of agreeableness was consistent with the previous survey for air pollution caused by industrialization [27] (S2 Appendix in S1 File). For all the other Big Five traits for both Future Generations and Yourself, the estimations were positive although they were relatively small and not always significant. For the Dark Triad, Machiavellianism and psychopathy showed significant negative contributions to *D* for both Future Generations and Yourself, implying that lower Machiavellianism and psychopathy contributed to higher *D*. Meanwhile, narcissism was estimated as positive for both Future Generations and Yourself, suggesting that higher narcissism contributed to higher *D*, although the effects were small. The two types of COVID-related variables were all estimated as significant (*p* < 0.001). *C*_*fear*_ was estimated as negative, suggesting that higher fear of COVID-19 contributed to larger *D*. This negative effect is consistent with the previous survey for air pollution caused by industrialization, where the nudging message was effective in datasets before the COVID-19 pandemic and became ineffective only in a dataset after the pandemic (Fig A in S2 Appendix in S1 File). Finally, *C*_*family*_, which measured the perceived threats of COVID-19 to respondents’ relatives and family, was positive for Future Generations and Yourself. One possible interpretation is that disposable plastics could also be regarded as helpful for preventing COVID-19 infection, which we did not realize when creating the designed messages. Because our designed messages said that packaging products in disposable plastics had contributed to improving hygiene, the message could promote this association, especially for respondents who thought that COVID-19 was a threat to their family and relatives. The value range for *C*_*fear*_ comprising seven questions on a five-point Likert scale was 7–35, whereas that for *C*_*family*_ comprising a single question on a five-point Likert scale was 1–5. Because the absolute values of the estimated coefficients for *C*_*family*_ were almost seven times larger than for *C*_*fear*_, and the signs were opposite, *C*_*family*_ could offset the negative effect of *C*_*fear*_ on average.**Qualitative survey**

After the intervention, we asked the respondents about how they felt reading the received messages using an open-ended question. Although the responses included neutral statements, such as ‘I have no idea’, ‘Nothing’, ‘None’, ‘NA’, ‘Not sure’, and ‘No comment’, there were both positive and negative responses in each country and each message group. We extracted representative messages for each group as examples, categorizing them as positive, neutral, and negative responses.

The typical positive responses across all three message groups tended to show that respondents changed their attitudes to viewing disposable plastics as less dangerous, as long as recycling is done properly. However, respondents mentioning the negative points of plastics as environmental destruction or dangerous to future generations tended to change their attitudes to seeing it as more dangerous. Most of the neutral responses mentioned both positive and negative points of plastics. Correspondingly, some respondents felt that the designed message was confusing (Table 7).

**Table 7:** Characteristic responses for CG.

	Japan	Canada	US
**Positive**	• *I do not think plastics are that bad if we can recycle them*. (Woman in her 30s, *D* = 2 for Yourself, 3 for Future Generations) • *They are used in medicines*, *food*, *and other things*. *I think there is no problem if we can expand post-processing and recycling after we use them*, *and can protect the environment*.(Man in his 50s, *D* = 3 for Yourself, 3 for Future Generations)	• *I like the fact that plastic can help reduce germs during traspertation*. *People just need to be more responsible when recycling*. *Furthermore*, *companies need to label these products better to state it can and should be recycled*. (Woman in her 30s, *D* = 2 for Yourself, 2 for Future Generations)	• *I am a huge recycler*. *I feel there is not enough education or resources for people to recycle*. *There is a ton of "wish"cycling going on*. *I feel every piece of plastic needs to be labeled with the number in the triangle to know for sure it can be recycled*. *Too many companies don’t tag plastic and therefore ends up in trash*. (Woman in her 50s, *D* = 4 for Yourself, 4 for Future Generations)
**Neutral**	• *Although it is good for human health*, *I think it is bad for the environment*. (Man in his 40s, *D* = 0 for Yourself, 0 for Future Generations) • *I guess garbage is increasing although germ infection is decreasing*. (Woman in her 60s, *D* = 0 for Yourself, 0 for Future Generations)	• *Everything has pros and cons*. *Disposable plastics has made our lives much easier*, *which is why it is so popular*. *Convenient and low costs*, *these attractive features made it very easy to overuse it*, *resulting in too much garbage and no one would take responsibility for after they enjoy the benefits*. (Woman in her 30s, *D* = 0 for Yourself, 0 for Future Generations) • *My initial reaction was around the negative impact to the environment*, *however this statement made me think about the benefits* (Woman in her 40s, *D* = 0 for Yourself, 0 for Future Generations)	• *There is a great deal of gray area around this issue including the negative effect plastics have on our environment vs the benefits received by them*. *There has been a push in recent years to limit our use of plastics and increase recycling*. *We need to find either another safer product to replace plastic or find a way to minimize the negative effects of plastic*. (Man in his 60s, *D* = 0 for Yourself, 0 for Future Generations) • *It’s very confusing to know what is the right thing to do*. (Man in his 60s, *D* = 0 for Yourself, 0 for Future Generations)
**Negative**	• *I wonder if it is true because it looks bad for the environment*. (Woman in her 30s, *D* = -2 for Yourself, -2 for Future Generations) • *Although recycling is better than before*, *marine pollution is serious*. (Man in his 40s, *D* = -1 for Yourself, -1 for Future Generations)	• *They say that plastic is dangerous for the environment* (Man in his 30s, *D* = -4 for Yourself, -4 for Future Generations) • *No because single use plastic is kill the environment just like any plastic would* (Woman in her 20s, *D* = -2 for Yourself, -2 for Future Generations)	• *It’s ruining the landfill and killing our oceans* (Woman in her 40s, *D* = -3 for Yourself, -3 for Future Generations) • *We need to start being environmentally friendly because we are killing the planet* (Man in his 30s, *D* = -3 for Yourself, -4 for Future Generations)

Note: Text in italics are the responses as they were written, including typographical errors. The responses for Japan were translated from Japanese

For CG, positive responses mentioned the benefits of plastics as well as the need for proper recycling. Neutral responses mentioned both benefits and risks, which sometimes made respondents determine their subjective judgements and resulted in no change in their attitudes. Negative responses mentioned the damage to the environment, especially to the ocean (Table 7).

The responses for T1 were similar to those for CG, in that the positive points focused on the benefits of plastics (Table 8). The positive response that was observed for this group and not observed for CG was the benefit from past generations (positive, in the US). The neutral responses for this group mentioned the feasibility of recycling and whether everyone would do so. There was a response in Canada mentioning that disposable plastics are not permitted, and their attitude did not change. The negative points included the need for alternatives to plastics (negative, in Japan and Canada) as well as danger to future generations (negative, in the US).

**Table 8:** Characteristic responses for T1.

	Japan	Canada	US
**Positive**	• *Because I heard plastics were a problem recently*, *my recognition was that they are bad things*. *But upon reading this sentence*, *I knew how plastic products are useful and they were not always bad*. (Man in his 20s, *D* = 3 for Yourself, 4 for Future Generations)	• *Disposable plastics are very beneficial for everyday life and so long as they are properly disposed of via recycling*, *the impact will hopefully be minimal on future generations*. (Woman in her 40s, *D* = 4 for Yourself, 4 for Future Generations)	• *It has been of great benefits since it was used in the past generation and it is still being used personally I think it has contributed positively to how society*. (Man in his 20s, *D* = 4 for Yourself, 4 for Future Generations) • *I try and be environmenrally conscious but not over reactive*. *I can see the benefits if plastic can be recycled*. (Woman in her 30s, *D* = 3 for Yourself, 4 for Future Generations)
**Neutral**	• *I think plastics are embedded in everyone’s life and the benefits are understood*. *Meanwhile*, *because people say that production of plastics should be reduced related to environmental problems including global warming*, *I think it is not negligible*. *I feel it is time to discuss optimized use of plastics*, *including recycling plastic products*.(Man in his 50s, *D* = 0 for Yourself, 0 for Future Generations)	• *plastics are both good and bad for us*.* *.* *.* *.*it help us in many practical ways in our everyday living however proper education*, *access*, *and handling of such items are not seriously done to prevent and manage the environmental crisis plastics pose* (Man in his 50s, *D* = 0 for Yourself, 0 for Future Generations) • *Disposable plastics I have not used for a long time as it is no longer permitted in Canada*, *I use recycled plastic or biodragible*. (Man in his 60s, *D* = 0 for Yourself, 0 for Future Generations)	• *I generally agree with this*. *We should recycle if possible but usually it’s not feasible to do so*. (Man in his 50s, *D* = 0 for Yourself, 0 for Future Generations) • *This only works and is safe if*.* *.* *. *EVERYONE recycles*. (Woman in her 50s, *D* = 0 for Yourself, 0 for Future Generations)
**Negative**	• *Because no alternative to useful plastics has been developed at this point*, *I think I have to segregate garbage and recycle plastics rigorously in everyday life to be responsible for the next generation*. (Woman in her 60s, *D* = -1 for Yourself, -1 for Future Generations)	• *I feel like we should teach new generations that the planet is not in good shape and that we need to help recycle to heal the world even if it’s a little it helps a lot* (Man in his 30s, *D* = -2 for Yourself, -2 for Future Generations) • *I think there needs to be a better way than using all that plastic to wrap products in*. (Woman in her 40s, *D* = -1 for Yourself, -4 for Future Generations)	• *Though disposable plastics have their benefits*, *they also are dangerous for future generations*. (Man in his 20s, *D* = -1 for Yourself, -1 for Future Generations)

Note: Text in italics are the responses as they were written, including typographical errors. The responses for Japan were translated from Japanese.

The responses for T2 were also similar to those for CG and T1, in that positive responses referenced the benefits of plastics and negative responses outlined concern about environmental destruction and damage to future generations (Table 9). The positive responses mentioned the support to future generations (positive, in Japan) or the relationship of the older and future generations (positive, in the US), different from CG. The most characteristic response for T2 was ‘easy to understand’ (positive, in Canada), suggesting that the additional illustration for this group helped respondents to interpret the message more easily (Table 9).

**Table 9:** Characteristic responses for T2.

	Japan	Canada	US
**Positive**	• *I think it is important to tackle the problem as the present generation for the next generations of children and grandchildren to live happily*. (Woman in her 60s, *D* = 3 for Yourself, 4 for Future Generations) • *My heart ached to see TV news on the terrible marine pollution by plastics that we are unconsciously using just because they are useful*. *Only thing I can do is to avoid receiving plastic spoons in supermarkets or to segregate recyclable garbage*, *but that would be great if everyone would think about contributions and benefits to future generations*. *I want to continue it*. (Woman in her 50s, *D* = 2 for Yourself, 4 for Future Generations)	• *easy to understand concept that should be applied* (Man in his 40s, *D* = 4 for Yourself, 4 for Future Generations) • *Makes a great deal of sense*. *I’ve been re-cycling plastics for years & don’t see why everyone shouldn’t do the same*. *We must provide a safe*, *healthy environment for our children & future generations*. (Woman in her 60s, *D* = 3 for Yourself, 3 for Future Generations)	• *I think this is a very informative message and we need to stress to the older generations the importance of recycling to protect the future for our grandchildren*. (Woman in her 60s, *D* = 3 for Yourself, 3 for Future Generations)
**Neutral**	• *I think the problem should be accepted to some extent as modern society though it is an important problem*. (Man in his 40s, *D* = 0 or Yourself, 0 or Future Generations) • *Though plastics have benefits that are helpful for safe transportation*, *there are drawbacks that they are not good for the environment*. *The sentence is very positive*, *but there are products that I wonder if they should be protected by plastic cases at all*. *I think we need to reconsider that point*. (Woman in her 30s, *D* = 0 or Yourself, 0 or Future Generations)	• *Showing the benefits of disposable plastics but realizing the negative impact on future generations* (Woman in her 30s, *D* = 0 for Yourself, 0 for Future Generations) • *This statement shows that there are pros and cons of different generations when it comes to disposable plastics* (Woman in her 20s, *D* = 0 for Yourself, 0 for Future Generations)	• *Disposable plastics have benefits*. *However we need to do more to protect the environment*. *Or find alternatives*. (Woman in her 60s, *D* = 0 for Yourself, 0 for Future Generations) • *I agree that plastic product use is far better than the disposal problems it produces*. (Man in his 40s, *D* = 0 for Yourself, 0 for Future Generations) • *I don’t think it’s a good idea because it provides more waste*. (Woman in her 40s, *D* = 0 for Yourself, 0 for Future Generations)
**Negative**	• *I think it is no good to burden younger generations now*. (Man in his 40s, *D* = -1 or Yourself, -1 or Future Generations) • *Plastic products have been beneficial*. *But it is important to reduce them because their compositions are not decomposed and persist perpetually*, *destroying natural environments*, *and having bad effects on plants and animals*. *I think we need to improve the present plastics fundamentally*. *I think they should be improved*, *and the compositions of plastics should be replaced with what decomposes in a few years in nature and has no bad effects on natural environments*. (Man in his 60s, *D* = -2 or Yourself, -1 or Future Generations)	• *Just because people in the past believed this doesn’t mean it’s right*. (Man in his 60s, *D* = -2 for Yourself, -3 for Future Generations) • *Growing access to recycling facilities wont magically make people put disposable plastics where they need to go*..*I think disposable plastics need to be phased out as soon as possible with better alternatives that are beneficial to us now*, *future generations and the environment* (Man in his 30s, *D* = -2 for Yourself, -2 for Future Generations)	• *I’ll believe that this statement is true there are good qualities to Plastics but they are ruining the Earth and the future Generations will have to pay the price there needs to be a replacement for Plastics and Mankind needs to stop producing plastic* (Man in his 50s, *D* = -2 for Yourself, -2 for Future Generations) • *Unfortunately*, *we now live in a “Disposable Society” whereby convenience trumps respect for the planet*. (Man in his 50s, *D* = -2 for Yourself, -2 for Future Generations)

Note: Text in italics are the responses as they were written, including typographical errors. The responses for Japan were translated from Japanese.

To investigate the effect of the COVID pandemic on the interventions, we focused on the respondents with high *C*_*family*_, which showed a positive contribution to the nudging message effects in the panel analysis (Table 10). This type of respondent tended to mention the benefits of plastics as a countermeasure for the COVID pandemic to maintain hygiene, changing their attitudes toward plastics as safer than perceived previously.

**Table 10:** Characteristic responses from respondents whose *C*_*family*_ was high.

Japan	Canada	US
*Disposable plastics are essential in this covid age… I want to rethink if they are really bad for the environment*. (Woman in her 60s, *D* = 1 for Yourself, 2 for Future Generations, *C*_*family*_ = 5, T1)	• *I try to recycle everything that I can and I wash all of my recyclables*, *but I notice younger people are too lazy to do this*. *I also heard a young woman saying she doesn’t recycle because it all winds up in the landfill anyway*. *Her husband drives a garbage truck*. *I worry that when youth talk that way w will never get rid of plastic*. *Also covid made it impossible to not use plastic bags*. (Woman in her 60s, *D* = 3 for Yourself, 2 for Future Generations, *C*_*family*_ = 4, T1)	• *Until the pandemic*, *we were trying to reduce use of plastics*. *Now we use more*. *It may keep us safer for now but I worry about the long term environmental impact*. (Woman in her 50s, *D* = 0 for Yourself, 1 for Future Generations, *C*_*family*_ = 4, T1)

Note: Text in italics are the responses as they were written, including typographical errors. The responses for Japan were translated from Japanese.

Although the present study focused on attitude changes toward disposable plastics in a single survey, durability of the intervention effects and actual behavioral changes are also important. Although we did not design a specific question for ascertaining these effects, some responses suggesting the long-term intervention effects were found in the open-ended question (Table 11). For example, a response obtained in Japan suggested that she would keep recycling after reading the T2 message. Similar responses were found in the US in T1 group. Even in Canada, where disposable plastic use was officially banned, responses suggested that the attitude changes caused by the intervention were not temporary. Of course, these responses do not guarantee that the respondents will behave as they said that they would. However, these responses did express their intention to contribute more to plastic recycling in the long term.

**Table 11:** Responses suggesting long-term intervention effects.

Japan	Canada	US
• *I had been usually disposing plastic products*, *but after reading this information*, *I thought that recycling is important to keep using plastic products*. *I have determined to cooperate for recycling from now on*. (Woman in her 30s, *D* = 0 for Yourself, 1 for Future Generations, T2)	• *I never thought too much about the benefits of disposable plastics*. *On reading the passage*. *I will give it more thought in the future*. (Man in his 30s, *D* = 0 for Yourself, 2 for Future Generations, T2)	• *I never thought of disposable plastics being such a benefit to myself and others*. *I will always recycle now*. (Woman in her 40s, *D* = 0 for Yourself, 1 for Future Generations, T1)

## Discussion

Although the DID effects for T1 emphasizing support across generations by textual information were negative, T2 with the additional illustrations, which presented essentially the same message as T1, showed significantly positive DID effects for Future Generations, and thus canceled the negative effects of T1 for Yourself. These results suggest that the T2 message rather than the T1 message should be used for information provision to increase positive attitudes toward disposable plastics. The T2 message increased perceived support from older generations and support to future generations, showing the strongest correlations with the message effects in all the three message groups.

Women showed a more positive attitude change on receiving the messages than men, which was consistent with our previous survey (Fig B in S2 Appendix in S1 File), whereas age had the opposite effect and older respondents showed a more positive reaction for Yourself than younger respondents. The contribution of sex was larger than that of age.

Although the target topic was not the same, our previous survey revealed that the DID effects of the designed messages would be weakened by the COVID-19 pandemic (S2 Appendix in S1 File). This tendency was replicated in the present survey too as expected, in that the scale for personal fear about COVID-19 showed a negative contribution to the designed message effects. Our designed message showed the intended effects of increasing positive attitudes toward disposable plastics, although the intervention effects were relatively weak compared with the previous survey due to the negative effects caused by personal fear about COVID-19.

In the meantime, respondents who were concerned about the COVID-19 effects on their family changed their attitudes toward being more positive about plastics, regardless of the type of the information provision, which is the opposite of the effect of personal fear about COVID-19. According to the responses in the open-ended question, this reaction may be caused by the information about plastic packaging maintaining hygiene, which drew more attention as a countermeasure in the COVID-19 pandemic than the risks of using plastic. These results suggest that COVID-19 has both positive and negative consequences on the message effects, depending on whether the fear is directed to the respondents themselves or their family.

Of the Big Five personality traits, agreeableness showed the largest contribution to the message effects, which was consistent with the previous survey (S2 Appendix in S1 File). We also sampled the Dark Triad for the present study. Low Machiavellianism and psychopathy also contributed to the message effects, which suggests that empathy is needed to be able to accept the message emphasizing support across generations. Although the contribution of narcissism to the message effects was weak compared with the other two Dark Triad facets, the positive contribution indicates that the perceived contribution to environmental problems could be rooted in the narcissistic motivation to do a “good thing”.

The DID effects by country were in the order US > Japan > Canada. While the message effects were affected both positively and negatively by the attitudes toward COVID-19 and were different by country, public policy on plastic use, which varies among the countries, could influence the message effects. On June 10, 2019, the Canadian prime minister declared that disposable plastic use was going to be banned in 2021 at the earliest [5], which was enacted as a regulation later [41]. Because the survey was conducted just after the first declaration, respondents may have felt that they had no choice in ending using plastics, which could have resulted in the lowest DID effects being obtained from Canada. Some states in the US already have regulations to ban disposable plastics, yet there is no nation-wide policy at this point. Although no policy to ban plastic use has been announced in Japan, a new regulation to promote plastic recycling was introduced [4] and plastic bags in supermarkets are no longer free as an economic intervention [3]. The effects of our designed messages could be decreased in the future if these interventions are strengthened.

Focusing on the most basic message for CG, the balance of positive and negative sentences may have mattered. Although we mentioned disposing plastics instead of recycling as a problem, the positive sentences mentioning the benefits were longer than the negative sentence. Incorporating more sentences mentioning risks caused by plastic recycling may decrease the message effects. To investigate the effects of this balance of positive and negative sentences, our future work will investigate the differential effects of such negative information by adding new message groups to be compared with the current message groups. There are known effects that can be caused by experimental settings, such as experimenter demand effects [46]. In particular, the presentation of our designed messages may have been slightly biased toward positive information about plastic recycling, which could unnecessarily promote more positive attitudes toward the provided information. In fact, the positive message effects were statistically significant in CG. Nonetheless, the effects caused by the information that could be biased to positiveness, if any, were included in both CG and T2. In other words, the DID effect was caused by the messages that were included in T2 but not in CG, namely, the emphasized presentation of support from relatives. Thus, the other unintended effects, such as the experimenter demand effects, were excluded by the DID evaluation, at least theoretically.

Although the message effects varied by segment and could be influenced by external factors, such as the COVID-19 pandemic or public policy, the proposed framework for information provision showed significant effects for multiple topics in multiple countries. Thus, messages emphasizing support from older generations with illustrations have universal effects on information provision as an intervention.

Our designed messages could be used for various promotional activities. One approach is to transform the messages into printed brochures and distribute them to the public for educational campaigns. This could be a straightforward application, which is similar to Home Energy Reports (HERs) [47], the effects of which were identified and widely applied to actual services, although the topic of HERs is promotion of energy conservation for residential sectors and different from our present study. Another approach is to use the messages in briefing sessions for residents when new recycling facilities are constructed, which may help to smooth negotiations about locating sites. However, because the effect sizes of these practical interventions for plastic recycling are currently unknown, identifying the effects in the realistic settings other than survey experiments for external validity is important future work.

## Conclusion

We conducted a randomized controlled trial using online surveys to investigate the effects of nudging messages in Japan, the US, and Canada. The messages were designed to increase positive attitudes toward disposable plastics. Highlighting support from older generations with illustrations showed significant intervention effects compared with the most basic textual information describing benefits and problems related to using disposable plastics.

The intervention effects were the largest in the US and smallest in Canada. Women changed their attitudes toward disposable plastics, seeing them as safer, on receiving any of the messages. For personality traits, respondents with higher agreeableness, lower Machiavellianism, lower psychopathy, and higher narcissism also changed their attitudes, viewing disposable plastics as safer. Attitudes toward COVID-19 showed different effects on the message effects: although personal fear about COVID-19 decreased the intervention effects, concern about the threat of COVID-19 to their family and relatives increased the effects.

The present study showed that the proposed framework (i.e., emphasizing support from older generations) could be easily and effectively used as an intervention for a wider variety of risk-related topics to increase positive attitudes. An example for future applications could include helping change attitudes toward unfamiliar new technologies, which tend to be judged as more dangerous than familiar technologies. This kind of risk-averse attitude could be a barrier to new technologies being adopted, which might decrease social welfare if the benefits were sufficiently larger than the risks. In this case, the proposed framework could be used if support from older generations, which may be difficult to define due to the lack of history of the new technology, were properly described.

## Supporting information

S1 File (DOC)

S1 Fig (ZIP)

S1 Data (CSV)
