# Supplementary material for: Cross-cultural comparison of nudging effects for environmental protection: A case-study of risk-averse attitudes toward disposable plastics
Source: PLoS One. 2022 Nov 3;17(11):e0277183. doi: 10.1371/journal.pone.0277183 (PMC9632847; doi:10.1371/journal.pone.0277183)
Supplement: S1 File — (DOC) [file pone.0277183.s001.doc]

**S1 Nomenclature**

COVID-19: Coronavirus disease of 2019

CG: Control Group

D: degree of post-intervention in attitude change toward disposable plastics

DID: difference-in-difference

HERs: Home Energy Reports

SDGs: Sustainable Development Goals

SE: Standard Error

T1: Treatment group 1

T2: Treatment group 2

US: United States

*Q*_pre_: pre-intervention attitudes toward disposable plastics

*Q*_post_: post-intervention attitudes toward disposable plastics’

**S1 Questionnaire**

**Questions for interventions**

- *Q*_pre_

What do you think of the impact of disposable plastics on daily lives for the following people?

|  | **Safe** | **Slightly Safe** | **Neutral** | **Slightly Dangerous** | **Dangerous** |
| --- | --- | --- | --- | --- | --- |
| **Future Generations** | 1 | 2 | 3 | 4 | 5 |
| **Yourself** | 1 | 2 | 3 | 4 | 5 |

- *Q*_post_

(One of the messages for CG, T1, and T2 is shown.**)**

**Please read the message about industrialization and air pollution and answer the question.**

Please answer the question below again. What do you think of the impact of disposable plastics on daily lives for the following people?

|  | **Safe** | **Slightly Safe** | **Neutral** | **Slightly Dangerous** | **Dangerous** |
| --- | --- | --- | --- | --- | --- |
| **Future Generations** | 1 | 2 | 3 | 4 | 5 |
| **Yourself** | 1 | 2 | 3 | 4 | 5 |

**S1 Table. Summary Statistics for *Q*_pre_ and *Q*_post_.**

| **Dataset** | **Question** | | **Mean** | **Standard Deviation** | **Min** | **Max** |
| --- | --- | --- | --- | --- | --- | --- |
| **Japan**  **(*n* = 4120)** | ***Q*_pre_** | **Future Generations** | 3.50 | 1.10 | 1 | 5 |
|  |  | **Yourself** | 2.71 | 1.00 | 1 | 5 |
|  | ***Q*_post_** | **Future Generations** | 2.98 | 1.19 | 1 | 5 |
|  |  | **Yourself** | 2.55 | 1.01 | 1 | 5 |
| **Canada**  **(*n* = 4120)** | ***Q*_pre_** | **Future Generations** | 3.76 | 1.35 | 1 | 5 |
|  |  | **Yourself** | 3.23 | 1.31 | 1 | 5 |
|  | ***Q*_post_** | **Future Generations** | 3.27 | 1.43 | 1 | 5 |
|  |  | **Yourself** | 2.84 | 1.31 | 1 | 5 |
| **US**  **(*n* = 4120)** | ***Q*_pre_** | **Future Generations** | 3.22 | 1.46 | 1 | 5 |
|  |  | **Yourself** | 2.74 | 1.37 | 1 | 5 |
|  | ***Q*_post_** | **Future Generations** | 2.76 | 1.45 | 1 | 5 |
|  |  | **Yourself** | 2.40 | 1.28 | 1 | 5 |

**Other questions**

- *Q*1

(One of the messages for CG, T1, and T2 is shown.**)**

Upon reading the passage below, do you think you are receiving benefits that increase your health and quality of everyday life from the following people?

(Your older relatives, including parents or grandparents)

| **I am benefitting** | **I am benefitting slightly** | **I am not benefitting much** | **I am not**  **benefitting** |
| --- | --- | --- | --- |
| 1 | 2 | 3 | 4 |

- *Q*2

(One of the messages for CG, T1, and T2 is shown.**)**

Upon reading the passage below, do you think "disposable plastic" is giving the benefits of increasing health and quality of everyday life to the following people?

(Your younger relatives, including children or grandchildren)

| **It has benefits** | **It has some benefits** | **It has few benefits** | **It does not**  **have benefits** |
| --- | --- | --- | --- |
| 1 | 2 | 3 | 4 |

- *Q*3

(One of the messages for CG, T1, and T2 is shown.**)**

Please let us know your impressions or thoughts when you read the passage below. Please answer freely.

- *Q*4

Please answer each of the questions below.

#Please answer about your children.

#Please answer 0 for all the questions if you have no children now.

1. How many children do you have?
2. How many children are living with you?
3. How many children are working in a paid job?

- *Q*5

Please let us know your parents’ current status about their living and working. (Please select as many as necessary for each question)

|  | **Father** | **Mother** | **None** |
| --- | --- | --- | --- |
| 1. **Living with you in the same house (or at the same site)** |  |  |  |
| 1. **Working in a paid job** |  |  |  |

- *Q*6

Since its onset, how has COVID-19 made you feel? Please select the answer that applies the most to you for each of the following items.

(COVID-19 threatens the lives of my family and relatives)

| **Very characteristic** | **Characteristic** | **Neutral** | **Uncharacteristic** | **Very uncharacteristic** |
| --- | --- | --- | --- | --- |
| 1 | 2 | 3 | 4 | 5 |

**S1 Appendix**

**Table A. Sample Ratios for Japan by Region.**

| **Region** | **Estimated area (km^2^)** * | **Collected samples** | |
| --- | --- | --- | --- |
|  |  | **Percentage (%)** | **Counts** |
| **Hokkaido** | 83,424 | 4.3 | 179 |
| **Tohoku** | 66,948 | 1.0 | 247 |
| **Kanto** | 32,434 | 5.3 | 1522 |
| **Chubu** | 66,807 | 1.8 | 680 |
| **Kinki** | 33,126 | 2.8 | 189 |
| **Chugoku** | 31,922 | 1.1 | 223 |
| **Shikoku** | 18,803 | 0.6 | 103 |
| **Kyushu** | 42,231 | 1.1 | 301 |
| **Okinawa** | 2283 | 0.7 | 27 |

Based on data retrieved from [1].

Regions are listed from north to south.

**Table B. Sample Ratios for Canada by Region.**

| **Region** | **Estimated area (km^2^)** * | **Collected samples** | |
| --- | --- | --- | --- |
|  |  | **Percentage (%)** | **Counts** |
| **British Columbia** | 944,735 | 15.4 | 635 |
| **Alberta** | 661,848 | 14.0 | 578 |
| **Saskatchewan** | 651,036 | 3.7 | 152 |
| **Manitoba** | 647,797 | 4.5 | 184 |
| **Ontario** | 1,076,395 | 46.7 | 1926 |
| **Quebec** | 1,542,056 | 7.2 | 298 |
| **New Brunswick** | 72,908 | 2.6 | 109 |
| **Nova Scotia** | 55,284 | 3.5 | 146 |
| **Prince Edward Island** | 5,660 | 0.5 | 20 |
| **Newfoundland and Labrador** | 405,212 | 1.7 | 69 |

Based on data retrieved from [2].

Regions are listed from west to east.

**Table C. Sample Ratios for the US by Region.**

| **Region** | **Estimated area (km^2^)** | **Collected samples** | |
| --- | --- | --- | --- |
|  |  | **Percentage (%)** | **Counts** |
| **Alabama** | 135,767 | 2.1 | 179 |
| **Alaska** | 1,723,337 | 0.3 | 31 |
| **Arizona** | 295,234 | 2.2 | 31 |
| **Arkansas** | 137,732 | 1.0 | 90 |
| **California** | 423,967 | 6.9 | 30 |
| **Colorado** | 269,601 | 1.7 | 19 |
| **Connecticut** | 14,357 | 1.3 | 46 |
| **Delaware** | 6446 | 0.4 | 69 |
| **District of Columbia (DC)** | 177 | 0.2 | 43 |
| **Florida** | 170,312 | 8.0 | 48 |
| **Georgia** | 153,910 | 3.9 | 242 |
| **Hawaii** | 28,313 | 0.4 | 212 |
| **Idaho** | 216,443 | 0.3 | 564 |
| **Illinois** | 149,995 | 3.5 | 344 |
| **Indiana** | 94,326 | 2.2 | 68 |
| **Iowa** | 145,746 | 1.2 | 32 |
| **Kansas** | 213,100 | 0.8 | 35 |
| **Kentucky** | 104,656 | 1.7 | 19 |
| **Louisiana** | 135,659 | 1.3 | 22 |
| **Maine** | 91,633 | 0.6 | 58 |
| **Maryland** | 32,131 | 1.7 | 59 |
| **Massachusetts** | 27,336 | 1.6 | 96 |
| **Michigan** | 250,487 | 2.8 | 291 |
| **Minnesota** | 225,163 | 1.4 | 50 |
| **Mississippi** | 125,438 | 0.9 | 32 |
| **Missouri** | 180,540 | 2.1 | 82 |
| **Montana** | 380,831 | 0.4 | 357 |
| **Nebraska** | 200,330 | 0.6 | 217 |
| **Nevada** | 286,380 | 1.3 | 48 |
| **New Hampshire** | 24,214 | 0.4 | 23 |
| **New Jersey** | 22,591 | 2.9 | 16 |
| **New Mexico** | 314,917 | 0.5 | 17 |
| **New York** | 141,297 | 7.7 | 60 |
| **North Carolina** | 139,391 | 3.5 | 101 |
| **North Dakota** | 183,108 | 0.1 | 29 |
| **Ohio** | 116,098 | 4.7 | 19 |
| **Oklahoma** | 181,037 | 1.5 | 30 |
| **Oregon** | 254,799 | 1.2 | 37 |
| **Pennsylvania** | 119,280 | 4.5 | 17 |
| **Rhode Island** | 4001 | 0.4 | 161 |
| **South Carolina** | 82,933 | 1.1 | 18 |
| **South Dakota** | 199,729 | 0.2 | 34 |
| **Tennessee** | 109,153 | 2.2 | 34 |
| **Texas** | 695,662 | 7.6 | 32 |
| **Utah** | 219,882 | 0.5 | 27 |
| **Vermont** | 24,906 | 0.2 | 24 |
| **Virginia** | 110,787 | 2.7 | 27 |
| **Washington** | 184,661 | 2.2 | 179 |
| **West Virginia** | 62,756 | 0.9 | 31 |
| **Wisconsin** | 169,635 | 2.0 | 31 |
| **Wyoming** | 253,335 | 0.1 | 90 |

Based on data retrieved from [3].

Regions are listed alphabetically.

**S2 Appendix**

This appendix describes results of information provision using the same experimental framework as the present study (Table A, B, Figure A, and B), regarding air pollution caused by industrialization based on four datasets: samples obtained on February 28 and March 1, 2019 in Japan (J-2019), samples obtained from February 25 to 27, 2020 in Japan (J-2020), samples obtained from February 27 to March 12, 2020 in Canada (C-2020), and samples obtained from February 27 to March 12, 2020 in the US (U-2020). All the results in this section are retrieved from:

Komatsu H, Tanaka N, Kubota H, Ohashi H. Searching for the universality of nudging: A cross-cultural comparison of the information effects of reminding people about familial support. PLOS ONE (in review).

**Table A. Correlation Coefficients Between Post-intervention Effect in Attitude Change Toward Air Pollution (*D*) and Perceptions of Benefiting From the Actions of Previous Generations.**

| **Dataset** | **J-2019** | | **J-2020** | | **C-2020** | | **U-2020** | |
| --- | --- | --- | --- | --- | --- | --- | --- | --- |
| **Group** | **Future Generations** | **Yourself** | **Future Generations** | **Yourself** | **Future Generations** | **Yourself** | **Future Generations** | **Yourself** |
| **CG + T1 + T2** | ***0.068 | ***0.085 | ***0.089 | ***0.084 | ***0.108 | ***0.102 | ***0.089 | ***0.098 |
| **CG** | 0.032 | 0.038 | 0.041 | 0.026 | **0.083 | ***0.091 | 0.035 | ^†^0.044 |
| **T1** | **0.087 | ***0.111 | ***0.106 | ***0.113 | ***0.113 | ***0.117 | ***0.097 | ***0.132 |
| **T2** | **0.067 | ***0.095 | ***0.119 | ***0.117 | ***0.108 | ***0.099 | ***0.103 | ***0.094 |

CG, control group; T1, treatment group 1; T2, treatment group 2.

^†^, *, **, *** Difference from zero with 90%, 95%, 99%, and 99.9% confidence, respectively.

**Table B. Correlation Coefficients Between Post-intervention Effect in Attitude Change Toward Air Pollution (*D*) and Perceptions That Younger Relatives are Benefiting From the Actions of Previous Generations.**

| **Dataset** | **J-2019** | | **J-2020** | | **C-2020** | | **U-2020** | |
| --- | --- | --- | --- | --- | --- | --- | --- | --- |
| **Group** | **Future Generations** | **Yourself** | **Future Generations** | **Yourself** | **Future Generations** | **Yourself** | **Future Generations** | **Yourself** |
| **CG + T1 + T2** | ***0.070 | ***0.071 | ***0.066 | *0.034 | ***0.162 | ***0.109 | ***0.133 | ***0.090 |
| **CG** | **0.085 | ***0.091 | 0.039 | 0.021 | ***0.165 | ***0.125 | ***0.099 | *0.063 |
| **T1** | *0.064 | ^†^0.051 | **0.081 | 0.028 | ***0.167 | ***0.098 | ***0.106 | *0.066 |
| **T2** | *0.058 | *0.068 | **0.077 | *0.054 | ***0.146 | ***0.103 | ***0.173 | ***0.124 |

CG, control group; T1, treatment group 1; T2, treatment group 2.

^†^, *, **, *** Difference from zero with 90%, 95%, 99%, and 99.9% confidence, respectively.


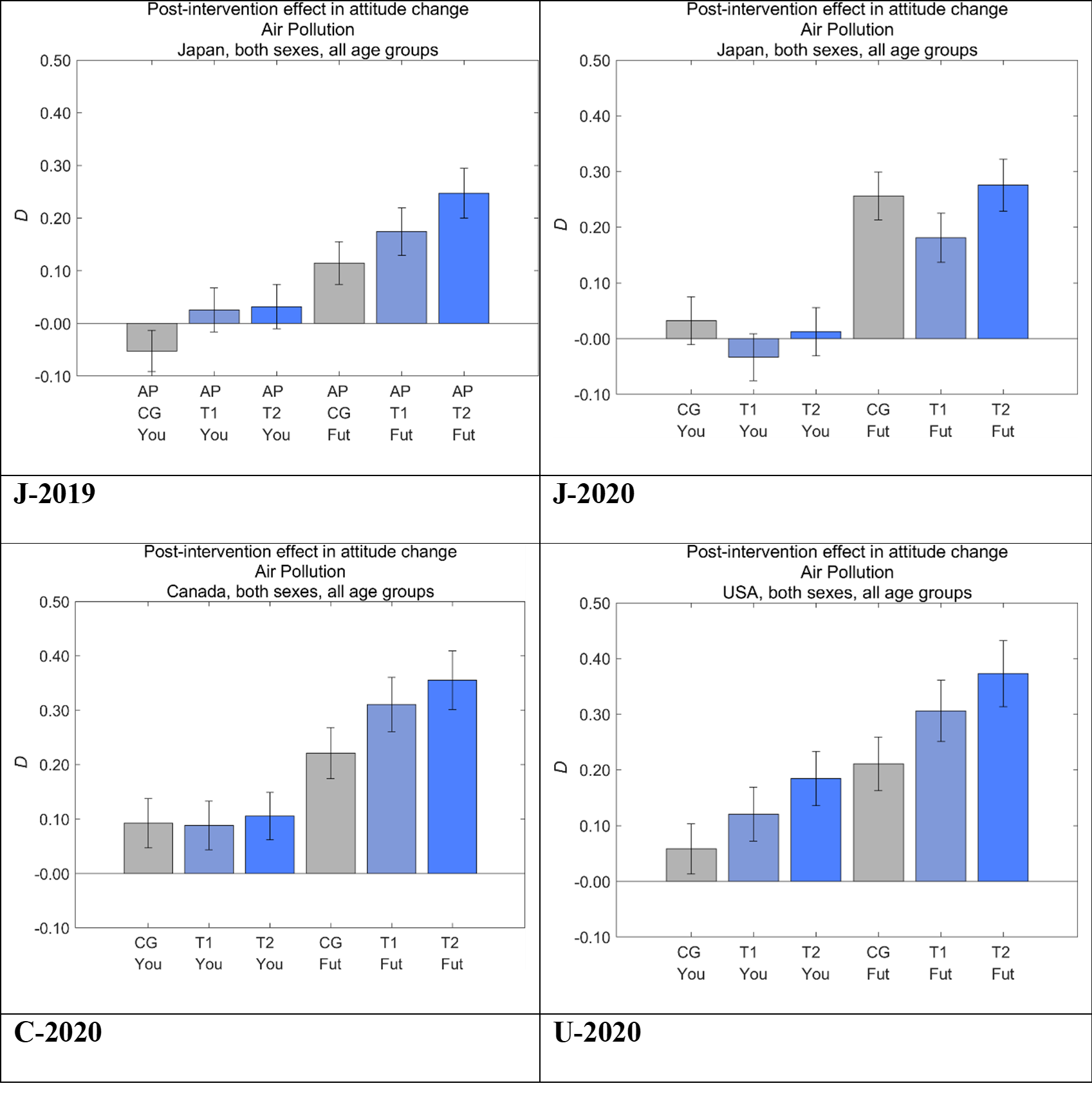


**Fig A. Post-intervention Effect in Attitude Change Toward Air Pollution (*D*).** Higher values on the vertical axis indicate lower perceived danger of air pollution. Error bars show 95% confidence intervals.


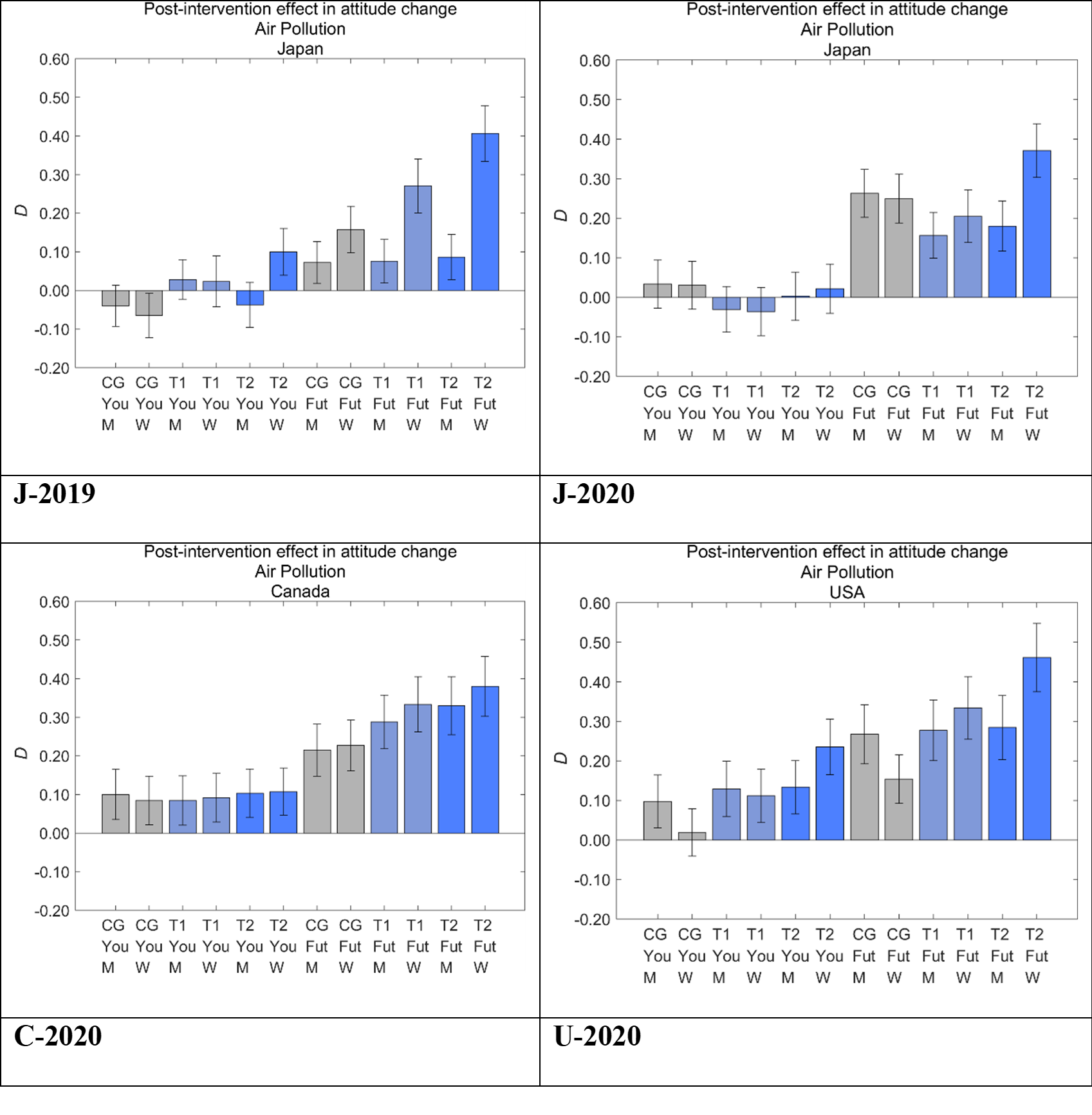


**Fig B. Post-intervention Effect in Attitude Change Toward Air Pollution (*D*) by Sex.** M, men; W, women. Error bars show 95% confidence intervals. Higher values on the vertical axis indicate lower perceived danger of air pollution.

**References**

- - - 1. Geospatial Information Authority of Japan. Estimated area by prefecture and city for 2020 [in Japanese]. 2019 [cited 8 April 2022]. Available from: https://www.gsi.go.jp/KOKUJYOHO/MENCHO/backnumber/GSI-menseki20200701.pdf
      2. Statistics Canada. Land and freshwater area, by province and territory. 2005 [cited 8 April 2022]. Available from: https://web.archive.org/web/20110524063547/http://www40.statcan.gc.ca/l01/cst01/phys01-eng.htm
      3. United States Census Bureau. United States Summary: 2010, Population and Housing Unit Counts, 2010 Census of Population and Housing. 2012 [cited 8 April 2022]. Available from: https://www2.census.gov/library/publications/decennial/2010/cph-2/cph-2-1.pdf
