# Supplementary figures and images for: Cross-cultural comparison of nudging effects for environmental protection: A case-study of risk-averse attitudes toward disposable plastics
Source: PLoS One. 2022 Nov 3;17(11):e0277183. doi: 10.1371/journal.pone.0277183 (PMC9632847; doi:10.1371/journal.pone.0277183)

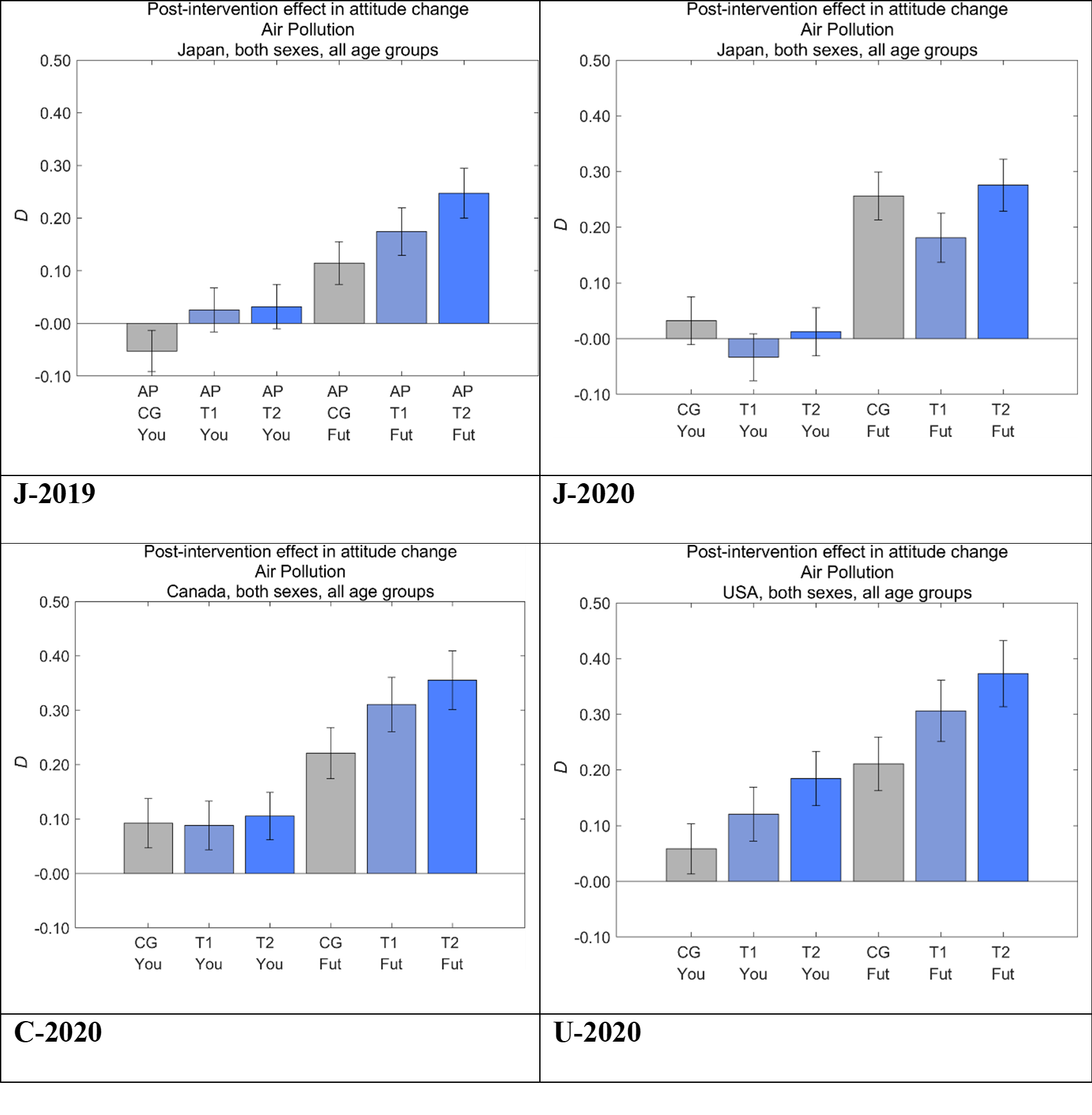

Supplement: S1 Fig — (ZIP) [file pone.0277183.s002.zip › S1A_Fig.tif]

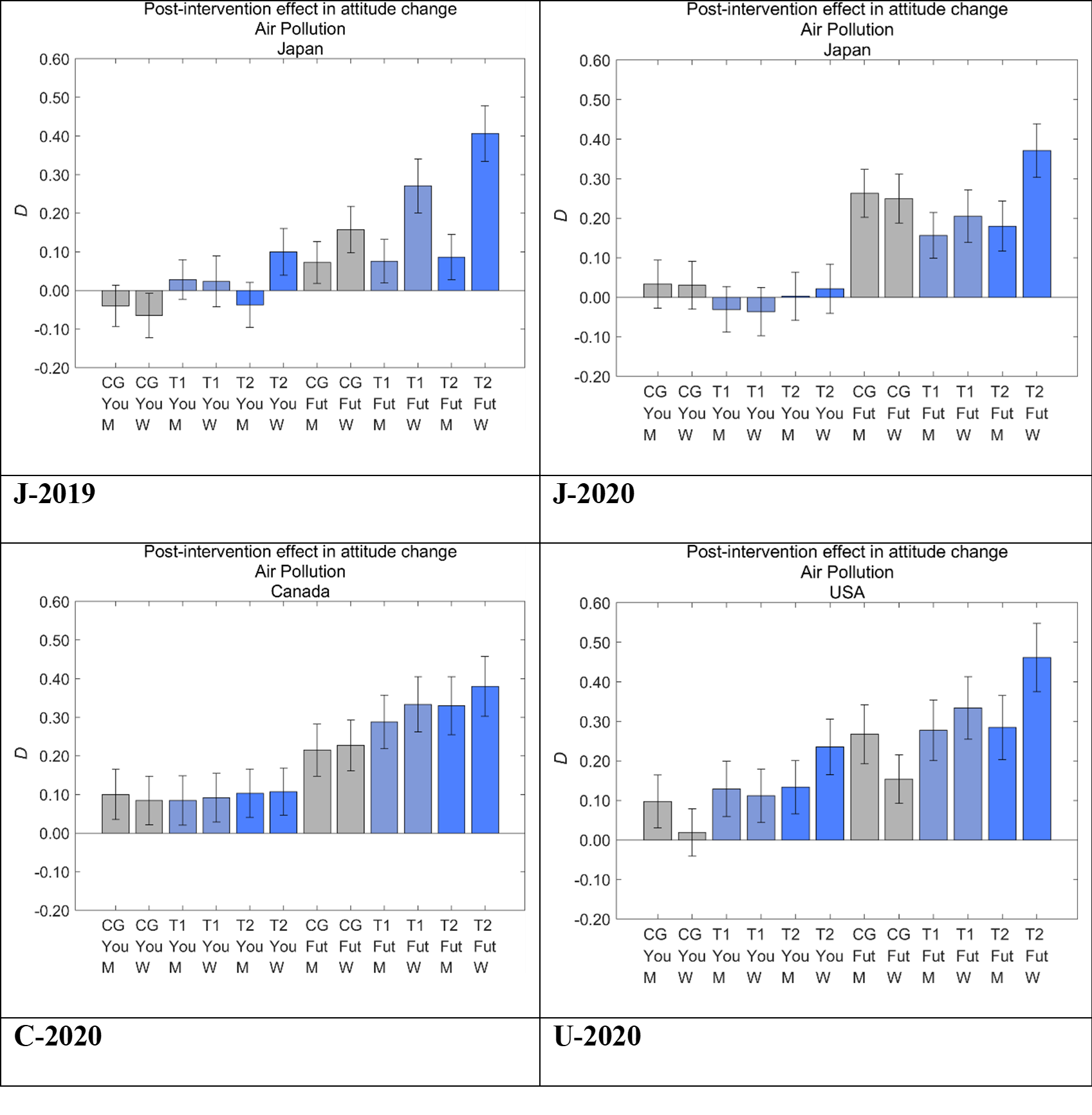

Supplement: S1 Fig — (ZIP) [file pone.0277183.s002.zip › S1B_Fig.tif]
